# The deficiency of chymase mast cell protease 4 exacerbates dextran sulfate sodium salt-induced colitis in mice and is associated with altered microbiota and metabolome profiles

**DOI:** 10.3389/fcimb.2025.1481927

**Published:** 2025-07-08

**Authors:** Zhiqiang Li, Xiaoyuan Kuang, Dashuang Mo, Magnus Åbrink, Ge Shan, Jin-ping Li, Can Yang, Yuexi Wang, Tao Shen, Weiwei Yu

**Affiliations:** ^1^ Department of Immunology, College of Basic Medicine, Guizhou Medical University, Guiyang, China; ^2^ The Key and Characteristic Laboratory of Modern Pathogen Biology, College of Basic Medicine, Guizhou Medical University; Department of Medical Parasitology, College of Basic Medicine, Guizhou Medical University, Guiyang, China; ^3^ Department of Medical Biochemistry and Microbiology, Uppsala University, Uppsala, Sweden; ^4^ Division of Anatomy, Physiology, Immunology and Patholog, Department of Animal Biosciences, Swedish University of Agricultural Sciences, Uppsala, Sweden; ^5^ Urologic Surgery Clinic, Tongren Municipal People’s Hospital, Tongren, China; ^6^ Department of Hepatobiliary Surgery, The Affiliated Drum Tower Hospital of Nanjing University Medical School, Nanjing, China

**Keywords:** ulcerative colitis, microbiota, chymase, exacerbation, metabolism

## Abstract

**Introduction:**

Ulcerative colitis (UC) is a chronic gastrointestinal disease characterized by symptoms of abdominal pain and diarrhea. Chymase is a serine protease released by the mast cells and is highly expressed in patients with UC. However, its role in disease pathogenesis remains poorly understood. In mice, mast cell protease-4 (MCP-4) is considered as the functional homolog of human chymase, sharing similar enzymatic activity and biological roles.

**Methods:**

To investigate the role of MCP-4 in UC, we induced colitis in Mcpt-4-deficient (Mcpt-4^∆Cre^) and littermate control (Mcpt-4^fl/fl^) mice using 2% dextran sulfate sodium salt (DSS) for 8 days followed by 2 days of water. After sacrifice, colon length was measured, and tissue samples were analyzed by histology (H&E and toluidine blue staining), qPCR, and protein assays. Colonic contents were collected for microbiota and metabolomics analysis.

**Results:**

The results show that the Mcpt-4-deficiency exacerbated colitis, as reflected by the significantly greater weight loss, higher histological scores, elevated levels of myeloperoxidase and most of determined cytokines. Furthermore, the Mcpt-4^∆Cre^ colitis mice displayed a distinct shift in colonic microbiota composition, notably with significantly increased abundance of bacteria (*Akkermansia* and *Turicibacter*), associated with potentially worsened inflammation in colitis models. In addition, metabolomic profiling revealed alterations in colonic metabolites involved in key Kyoto Encyclopedia of Genes and Genomes (KEGG) pathways, including NF-kappa B signaling, Th1 and Th2 cell differentiation.

**Discussion:**

These findings reveal that the mouse chymase MCPT-4 plays important roles in maintaining the intestinal homeostasis during colitis, potentially through regulation of colonic cytokines, microbial and metabolic networks.

## 1 Introduction

Ulcerative colitis (UC) is a form of inflammatory bowel disease, and patients with UC have an increased risk of developing colorectal cancer (Voelker, 2024). UC occurs more common in young adults, which not only seriously lowers the life quality of patients but also increases the economic burden for society (Armuzzi and Liguori, 2021; Burisch et al., 2023). Because of the unclear pathogenesis and difficulty in treatment, UC has been listed by the World Health Organization as one of the difficult-to-treat diseases (Guo et al., 2024). The pathogenesis of UC is complicated, being a consequence of several interacting factors, such as genetic susceptibility, immune responses, imbalanced gastrointestinal microbiota, and metabolism (Ungaro et al., 2017). Gut microbiota is essential for digestion, nutrient absorption, and the maintenance of immune homeostasis (Glassner et al., 2020; Hirten and Sands, 2021). The alteration of bacterial composition or metabolites in gut was demonstrated to influence the inflammatory degree of UC (Yuan et al., 2021; Gowen et al., 2023). Transplantation of fecal bacteria collected from healthy donors could reduce the level of dextran sulfate sodium salt (DSS)-induced colitis in mice (Yang et al., 2023), indicating the functional roles of gut microbiota in colitis mice. Shifts in the gut microbiota could lead to changes in bacterially derived metabolites, which, in turn, modulates the mucosal immune environment and influences susceptibility to DSS-induced colitis (Yang et al., 2022; Yang et al., 2023). The metabolites tryptophan and short-chain fatty acids had been evidenced to exert an essential role in maintaining the immune homeostasis and the integrity of the intestinal barrier (Mann et al., 2024; Schirmer et al., 2024). For instance, in DSS-induced colitis, the cytoplasmic aryl hydrocarbon receptor, a transcription factor activated by binding xenobiotic ligands such as tryptophan metabolites and short-chain fatty acids, plays a regulatory role in regulating colonic inflammation (Pernomian et al., 2020).

Chymase is a serine protease primarily expressed by mast cells. In human, chymase is produced by mast cells expressing both chymase and tryptase (Pejler, 2020). In mouse, several different chymases are expressed, i.e., mast cell protease (MCP)-1, MCP-2, MCP-4, MCP-5, and MCP-9, of which MCP-4 has similar properties with human chymase in substrate cleavage profile, tissue distribution, and proteoglycan binding (Pejler, 2020). The chymase Mcpt-4, released by activated mast cells, exerts diverse biological functions through its enzymatic activity, including extracellular matrix remodeling, regulation of epithelial barrier integrity, and modulation of inflammatory responses (Atiakshin et al., 2019). *In vitro*, mouse MCP-4 was found to be pro-inflammatory evidenced by cleaving the tight junctions among endothelial or epithelial cells, causing the distortion of permeability (Pejler, 2020), while the degradation of some inflammatory factors including heat shock protein 70, HMGB1, and interleukin-33 (IL-33) showed the potential protective role of chymase in danger-induced inflammation (Roy et al., 2014). *In vivo*, the lack of Mcpt-4 gene in mice showed the altered intestinal barrier structure and function, as evidenced by reduced intestinal epithelial cell migration and enhanced intestinal permeability (Groschwitz et al., 2009). In addition, chymase MCP-4 was demonstrated to be involved in intestinal disorders. For instance, the deficiency of Mcpt-4 shifted the inflammatory responses of adult mature mice infected with *Giardia* by regulating the intestinal cytokines such as IL-2, IL-4, IL-5, IL-6, IL-10, and IL-25 (Li et al., 2020). In both UC patients and mouse models of colitis, the number of chymase-positive mast cells was increased. Moreover, treatment with a chymase inhibitor has been shown to alleviate colonic inflammation in DSS-induced colitis in mice (Andoh et al., 2006; Stasikowska-Kanicka et al., 2012; Liu et al., 2016), indicating that chymase may play a critical role in the pathogenesis of colitis.

To investigate the potential roles of the chymase mMCP-4 in colitis, a disease model was established with mice lacking gene Mcpt-4 (Mcpt-4^ΔCre^) along with control mice (Mcpt-4^fl/fl^). The analysis of phenotypic indicators, including colon length, colonic histological score, and inflammatory cytokine levels, as well as shifts in the microbiota and metabolism, indicates that Mcpt-4 played important roles in the pathology of experimentally induced colitis.

## 2 Materials and methods

### 2.1 Mice

The mast cell protease 4 (Mcpt-4) (Gene ID: 17227) conditional knockout mouse model, on the C57BL/6J genetic background, was custom made at Cyagen (Shanghai) using a targeting vector carry loxP sites in intron 1 and intron 4 and a Neomycin-resistance cassette flanked by self-deletion anchors (SDAs) next to the loxP site in intron 1. After Neomycin deletion, the floxed Mctp4 mouse strain was crossed with the transgenic mouse model carrying a tamoxifen-inducible Cre-recombinase CAGGCre-ER™ (https://www.jax.org/strain/004682). The breeding pairs received from Cyagen were either Mcpt-4^flox/flox^ or Mcpt-4^flox/flox/Cre^ (Mcpt-4^ΔCre^) (**Supplementary Figure 1a**). The mice were kept in the Department of Immunology, College of Basic Medicine, Guizhou Medical University. All mice were treated humanely and housed in an enriched environment. Permission (No. 2200084) for experiments with mice was granted by Guizhou Medical University’s Experimental Animal Center.

### 2.2 Genotyping of Cre tool gene

Littermate mice were produced by breeding and mice with the transgenic Cre-tool CAGGCre-ER™ were identified by PCR, using the primers, Forward 5′-CAA GGT CCA ACT AAC TCC CTT TGT GCT CC-3′ and Reverse 5′-GGT GAT CTC CAG ATG GGC CAT GTAAGG GCG-3′, yielding the expected 482-bp PCR product. To produce the mice needed for the experimental setup of the colitis disease model, the littermate mice carrying the transgene (i.e., Mcpt-4^flox/flox/Cre^) were intraperitoneally injected daily for 5 days with tamoxifen (Sigma) 20 mg/kg dissolved in maize oil (**Supplementary Figure 1b**).

### 2.3 Experimental mouse colitis disease model

To induce acute experimental colitis, 10- to 12-week-old littermate Mcpt-4^fl/fl^ and Mcpt-4^ΔCre^ mice received 2% DSS (36,000–50,000 Da, 0216011050, MP Bio) in water for 8 days followed by sterile water for 2 days. Body weight was recorded daily, and the weight changes of the two groups were pooled and evaluated from two independent experiments (**Supplementary Figure 2**), where the initial weight at day zero was used for normalization to the body weight of the following experiment days. On day 10, all mice were sacrificed and colon tissues collected from experiment #1 were separated for histological scoring and morphological staining, whereas colon tissues sampled from experiment #2 were used for evaluation of chemokine and cytokine levels as well as the analysis of microbiota and metabolism.

### 2.4 Detection of Mcpt-4 expression

For the detection of Mcpt-4 expression in colon of experimental mice, the RNA in tail tissue samples was purified according to the manufacturer’s protocol (Cat. No. 9765, MiniBEST Universal Genomic DNA Extraction Kit Ver,5.0, Takara). The Mcpt-4 expression was identified with qPCR using the primers, Forward 5′-GTAATTCCTCTGCCTCGTCCTT-3′ and Reverse 5′-GACAGGATGG-ACACATGCTTT-3′. Maxima SYBR Green/ROX qPCR Master Mix (Thermo Fisher Scientific) was used for the qPCR, and the expression of GAPDH was used for normalization according to the guidelines of AB Applied Biosystems (Step One Plus Real Time PCR systems) (**Supplementary Figure 1c**).

### 2.5 Colon sampling and morphological assessment

After the measurement of colon lengths of experimental mice, 1 cm of the collected colon was fixed with 10% formalin overnight then embedded in paraffin and cut into 4- to 5-µm tissue sections. The sections were mounted on slides and stained with acidic toluidine blue or hematoxylin and eosin (H&E). Colon lengths were compared among groups, and those from DSS-treated mice normalized by subtracting the average colon length of their respective water-treated genotype controls were statistically calculated. Pathological changes were assessed by light microscopy, and the numbers of mast cells in the whole tissue sample were counted in each mouse colon section. Histological alterations were observed by optical microscopy after staining and scored as described in Refs (Liu et al., 2024; Zhang et al., 2024), with summarized results presented in **Table 1**.

**Table 1:** Histological scoring criteria.

Score	Muscle thickening	Crypt damage	Cellular infiltration	Inflammation
0	normal	None	normal	none
1	mild	Basal 1/3 damaged	mild	slight
2	moderate	Basal 2/3 damaged	moderate	moderate
3	extensive	Only surace epithelium intact	extensive	severe
4	–	Entire crypt and epithelium lost	–	–

### 2.6 Detection of colonic myeloperoxidase, Mcpt-4, and cytokines

In brief, the colon tissues were stored in liquid nitrogen and smashed into tissue powder, of which 50 mg was suspended in Hanks’ balanced salt solution and then spun down by 15,000 × *g* centrifugation for a half hour at 4°C to an insoluble pellet. Using the manufacturer’s protocol, the colonic concentrations of myeloperoxidase (MPO) in the obtained supernatant were determined using mouse MPO (Cat. No. ab275109, Abcam) and Mcpt-4 (Cat. No. DEIA-FN890, Creative Diagnostics) ELISA developmental kits. As for the cytokine detection, mice with weight loss closing to the average trend were picked up and the Luminex multiplex assay was performed to detect the colonic chemokines KC/CXCL1, MCP-1/CCL2, MIP-1a/CCL3, MIP-1b/CCL4, RANTES/CCL5, Eotaxin/CCL11, G-CSF, and GM-CSF; proinflammatory cytokines IL-1a, IL-1b, TNF-a, and IL-6; Th1 cytokines IFN-*γ*, IL-2, and IL-12; Th2 cytokines IL-4, IL-5, IL-9, IL-10, and IL-13; Th17 cytokine IL-17A; and IL-12p40 using the kit (LX-MultiDTM-23, Shanghai LabEx Biotech).

### 2.7 16S rRNA gene sequencing and microbiome analysis

Total DNA was isolated from colon collected from experimental mice using the DNeasy^®^ PowerSoil^®^ Pro Kit (QIAGEN, USA) according to the supplier’s protocol. The 16S rRNA V3–V4 hypervariable regions of fecal and colonic bacteria were amplified with the extracted genomic DNA using primers 338 Forward (5′-ACT CCT ACG GGA GGC AGC AG-3′) and 806 Reverse (5′-GGA CTA CHV GGG TWT CTA AT-3′). The PCR products were examined by gel electrophoresis and purified with the AxyPrep DNA Gel Extraction Kit (Axygen Biosciences, Axygen, USA). Sequence libraries were generated using the NEXTFLEX^®^ Rapid DNA-Seq Kit. Sequencing was performed using the Illumina MiSeq PE300/NovaSeq PE250 platform (Illumina, San Diego, USA) according to the manufacturer’s instructions (Majorbio, Shanghai, China). The quality of the original sequence data was assessed using fastp (https://github.com/OpenGene/fastp, version 0.20.0) and spliced using FLASH (https://github.com/OpenGene/fastp, version 1.2.7). Operational taxonomic units (OTUs) were clustered based on a 97% similarity threshold by UPARSE (version 7.1). The analysis of taxonomy for each sequence was performed by comparing the Ribosomal Database Project (RDP) Classifier against the 16S rRNA database. The α-diversity of bacteria was calculated based on the OTUs using the ACE index. Differences in bacteria based on relative abundance at the genus levels were analyzed by the Kruskal–Kallis *H* test to compare four groups. Principal component analysis (PCA) or principal coordinates analysis (PCoA) of Bray–Curtis distance was utilized to estimate the β-diversity of the bacterial data. The bacteria at the genus level were counted and the overlap of bacteria among groups was shown with a Venn diagram. The average abundance of the top 10 bacteria at the genus level in four groups was shown with a histogram (community barplots) to visualize genus-level relative abundance profiles for descriptive purposes. Linear discriminant analysis effect size (LEfSe) tools were utilized to perform LEfSe analyses with a threshold of 2 on the logarithmic linear discriminant analysis (LDA) score (Baek et al., 2023). Associations between gut bacteria and colonic cytokines were tested using Spearman correlation analysis. The datasets presented can be found in online repositories (NCBI, PRJNA1150579). The data were analyzed through the free online platform of majorbio cloud platform (cloud.majorbio.com).

### 2.8 Metabolite extraction and analysis

The detailed protocol was described in the **Supplementary Materials**. In brief, 50 mg of colonic content was ground in extraction solution for metabolite extraction. Then, the samples were treated with low temperature twice; after the centrifugation, the supernatant was transferred to the injection vial for metabolite analysis. The LC-MS/MS analysis of the sample was carried out on a Thermo UHPLC-Q Exactive HF-X system. The raw data were processed by Progenesis QI (Waters Corporation, Milford, USA) software, and a three-dimensional data matrix (sample information, metabolite name, and mass spectral response intensity) in CSV format was exported. Meanwhile, the variables of QC samples with relative standard deviation (RSD) > 30% were excluded, and log10 logarithmic zed was used to obtain the final data matrix for subsequent analysis. Then, the R package “ropls” (Version 1.6.2) was used to perform PCA and orthogonal least partial squares discriminant analysis (OPLS-DA) to evaluate the group separation and seven-cycle interactive validation to evaluate the stability of the model. The metabolites with fold change (log_2_FC) > 0 threshold, variable importance in the projection (VIP) > 1, and *p* < 0.05 were determined as significantly different metabolites based on the VIP obtained by the OPLS-DA model and the *p*-value generated by Student’s *t*-test (Liu et al., 2021; Yu et al., 2023). The metabolites were counted and the overlap among groups was shown with a Venn diagram. Differential metabolites among two groups were mapped into their biochemical pathways through metabolic enrichment and pathway analysis based on the KEGG database. These metabolites could be classified according to the pathways they are involved in or the functions they performed. Enrichment analysis was used to analyze a group of metabolites in a functional node, whether appearing or not. The principle was that the annotation analysis of a single metabolite could develop into an annotation analysis of a group of metabolites. The Python package “scipy.stats” (https://docs.scipy.org/doc/scipy/) was used to perform enrichment analysis to obtain the most relevant biological pathways for the experimental setup. The datasets presented can be found in online repositories (figshare: https://figshare.com/s/693809d934f451ac0b0e). The data were analyzed through the online platform of the majorbio cloud platform (cloud.majorbio.com).

### 2.9 Spearman correlation analysis

**Figure 1:**
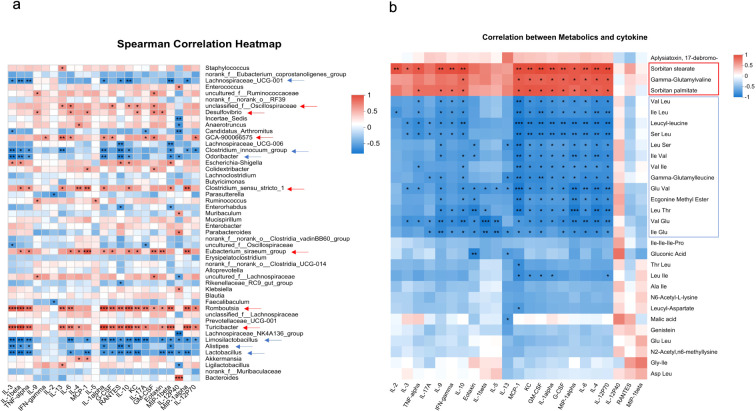
Correlation of colonic cytokines with microbiota or metabolite. Heatmaps showing Spearman correlation coefficients between colonic cytokine levels and gut microbiota at the genus level **(a)** or colonic metabolites **(b)** across all four experimental groups. Red indicates a positive correlation and blue indicates a negative correlation. The intensity of the color corresponds to the strength of the correlation. Asterisks indicate statistical significance: *p < 0.05, **p < 0.01, and ***p < 0.001. Arrows highlight microbial genera or metabolites that show significant correlations with more than five cytokines.

Spearman correlation analysis is a non-parametric statistical method used to measure the strength and direction of the monotonic relationship between two variables. Here in our work, Spearman correlation analysis was performed using R version 3.1.1 to evaluate the relationship between the detected inflammatory cytokines and microbiota composition or metabolic parameters in experimental mice. For each analysis, raw data for cytokines, microbiota, and metabolites from the same mouse were uploaded to the Majorbio platform, where Spearman’s rank correlation coefficients were calculated. The resulting heatmap in **Figure 1** visualizes these correlations. Color intensity indicates the strength and direction of the correlation (red for positive correlations and blue for negative correlations). Color depth corresponds to the correlation coefficient value, with darker shades indicating stronger correlations.

### 2.10 Statistical analysis

Statistical analysis of the experimental data involved either ordinary one-way ANOVA or a *t*-test with Welch’s correction using GraphPad Prism software, as presented in each figure legend. For microbiota analysis, α-diversity was compared between groups using the Kruskal–Wallis *H* test. The relative abundance of bacterial genera with statistically significant differences was determined using Welch’s *t*-test. *p*-values <0.05 were considered to be statistically significant.

## 3 Results

### 3.1 The chymase mouse mast cell-specific protease-4 was highly expressed in DSS-induced colitis mice

**Figure 2:**
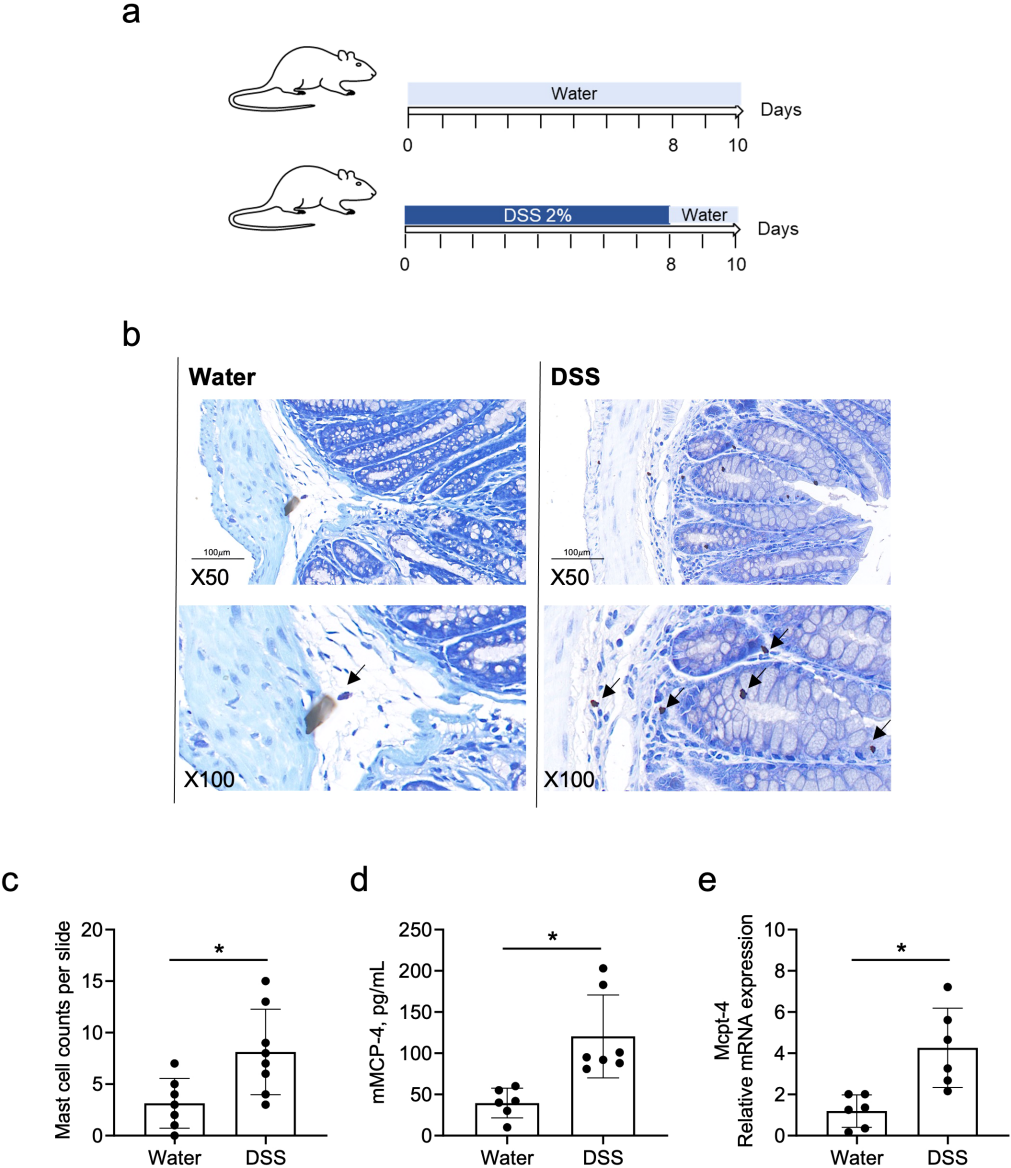
The mouse chymase mast cell protease 4 is highly expressed in DSS-induced colitis mice. Age-matched C57BL/6 mice were used to establish the DSS-induced colitis mouse model, as illustrated in **(a)**. On day 10, mice from experiment #1 were sacrificed and mast cell numbers were counted, ×50 and ×100 magnification **(b)** and statistically analyzed **(c)**, in colon tissue stained with toluidine blue. The levels of Mcpt-4 in the colon tissue was determined by ELISA **(d)** and qPCR **(e)**. Data are shown as mean ± SE and statistical analysis was conducted by Welch’s *t*-test with significant difference presented as *p < 0.05.

It has been reported that chymase-positive mast cell numbers increased in the colorectal tissue of UC patients and in DSS-induced colitis (Andoh et al., 2006; Cho et al., 2011). To verify whether chymase mMCP-4 is involved in colitis, we established the colitis mouse model, as illustrated in **Figure 2a**. The number of mast cells in colon tissue was counted and the levels of both mMCP-4 protein and Mcpt-4 gene were examined. Compared to mast cell numbers in naïve mice, mice with colitis had significant increase in numbers of mast cells (**Figures 2b, c**), which was reflected by a significant increase of mMCP-4 levels (**Figures 2d, e**), suggesting that Mcpt-4 may play an important role in colitis.

### 3.2 The deficiency of Mcpt-4 exacerbated colitis in mice

**Figure 3:**
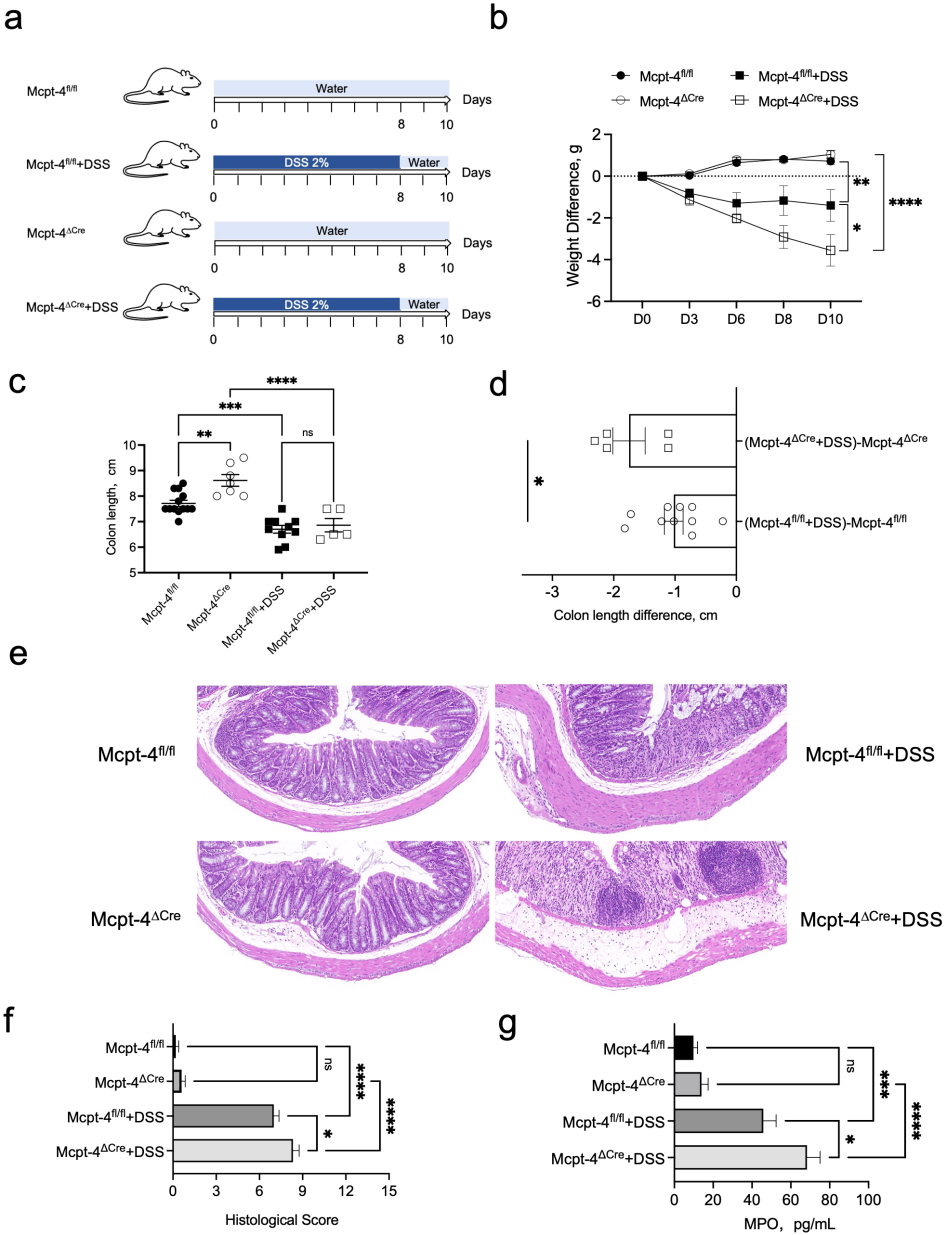
Mcpt-4 deficiency exacerbates DSS-induced colitis. Groups of age-matched Mcpt-4^fl/fl^ and Mcpt-4^ΔCre^ C57BL/6 mice (experiment #1, *N* ≧ 5) were treated with 2% DSS to induce colitis **(a)**. The body weight was scored every 2/3 days, and weight change normalized to the day zero weight was compared; the significance among groups on day 10 is shown **(b)**. Colonic length was measured in the Mcpt-4^fl/fl^, Mcpt-4^fl/fl^ +DSS, Mcpt-4^ΔCre^, and Mcpt-4^ΔCre^ +DSS mice groups **(c)** and colon lengths from DSS-treated mice normalized by subtracting the average colon length of their respective water-treated genotype controls (Mcpt-4^ΔCre^ or Mcpt-4^fl/fl^) were compared **(d)**. Representative hematoxylin and eosin (H&E)-stained colon sections for each group are shown **(e)** and histological scores were assigned based on inflammation, crypt damage, and epithelial disruption in colon sections **(f)**. The level of myeloperoxidase (MPO) was determined in 100 mg of colon tissue **(g)**. Data are shown as mean ± SE, and statistical analysis was conducted by ordinary one-way ANOVA for **(b, c, f, g)** and Welch’s *t*-test for **(d)** with significant difference presented as *p < 0.05, **p < 0.01, ***p < 0.001 and ****p < 0.0001; ns, no significance.

To investigate the potential role of mMCP-4 in colitis, we employed the Mcpt-4 gene knockout (Mcpt-4^ΔCre^) mice (**Supplementary Figure 1**) and induced colitis with DSS as illustrated in **Figure 3a**. The results show that DSS-induced colitis led to significantly greater weight loss (**Figure 3b**), shorter colon length (**Figures 3c, d**), higher pathological scores (**Figures 3e, f**), and increased MPO levels (**Figure 3g**) in the Mcpt-4^ΔCre^ colitis mice as compared with the control Mcpt-4^fl/fl^ colitis mice. These reveal that the deficiency of Mcpt-4 led to further colon tissue damage and inflammation in colitis mice, suggesting that the Mcpt-4 deficiency exacerbates colitis.

### 3.3 The Mcpt-4 defect enhanced production of the related chemokines and pro-inflammatory cytokines in colitis mice

**Figure 4:**
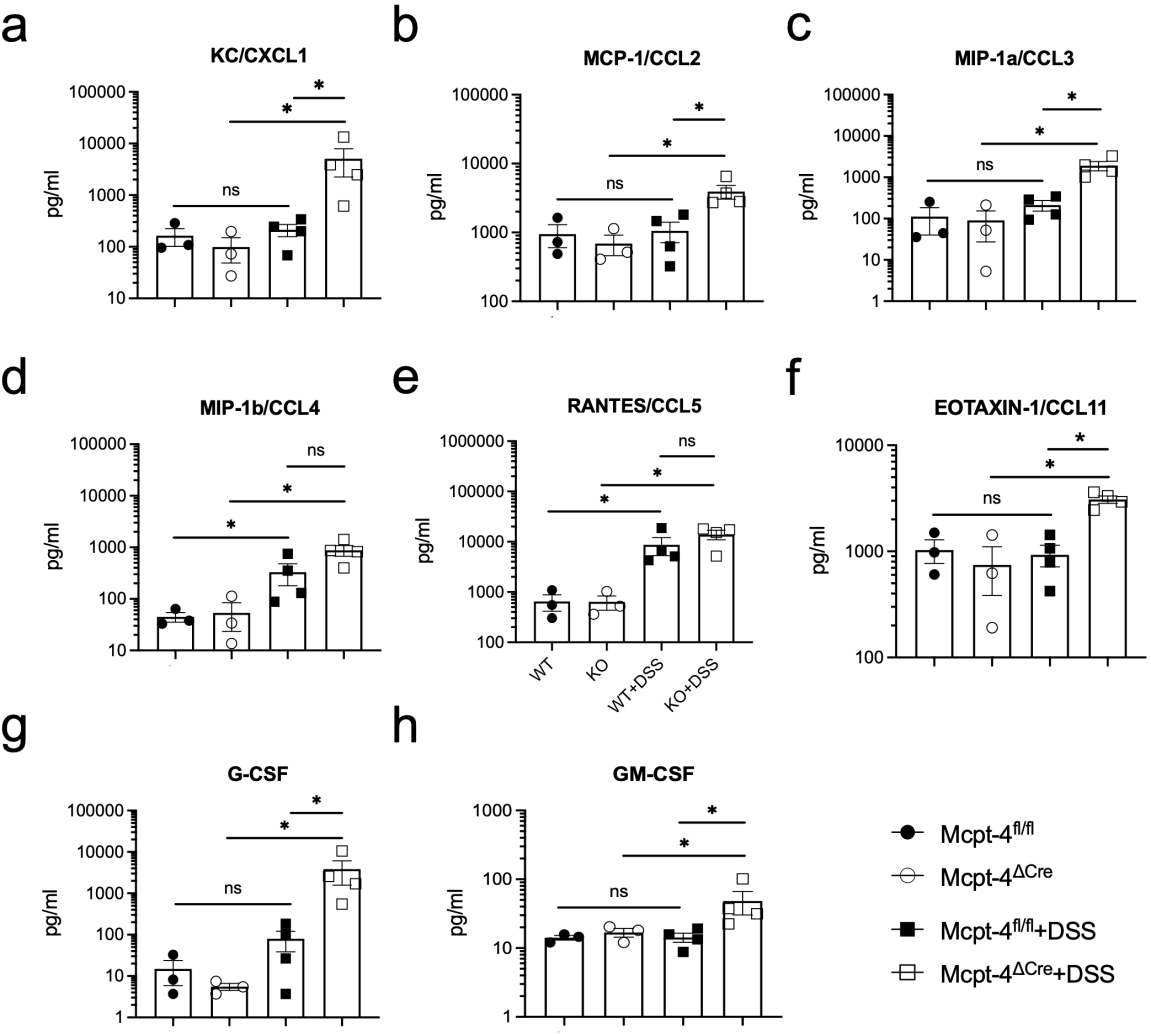
Mcpt-4 deficiency results in higher colonic chemokine production in mice with colitis. Chemokine levels of CXCL1 **(a)**, CCL2 **(b)**, CCL3 **(c)**, CCL4 **(d)**, CCL5 **(e)**, CCL11 **(f)**, G-CSF **(g)**, and GM-CSF **(h)** were examined by the Luminex multiplex assay in colon tissue extracts from the Mcpt-4^fl/fl^, Mcpt-4^ΔCre^, Mcpt-4^fl/fl^ +DSS, and Mcpt-4^ΔCre^ +DSS mice groups (*n* ≧ 3) from experiment #2, duplicate for each sample. Data are shown as mean ± SE and statistical analysis was conducted by Welch’s *t*-test with significant difference presented as *p < 0.05, ns, no significance. Note that there is no significant difference between the non-colitis Mcpt-4^fl/fl^ and Mcpt-4^ΔCre^ control groups.

**Figure 5:**
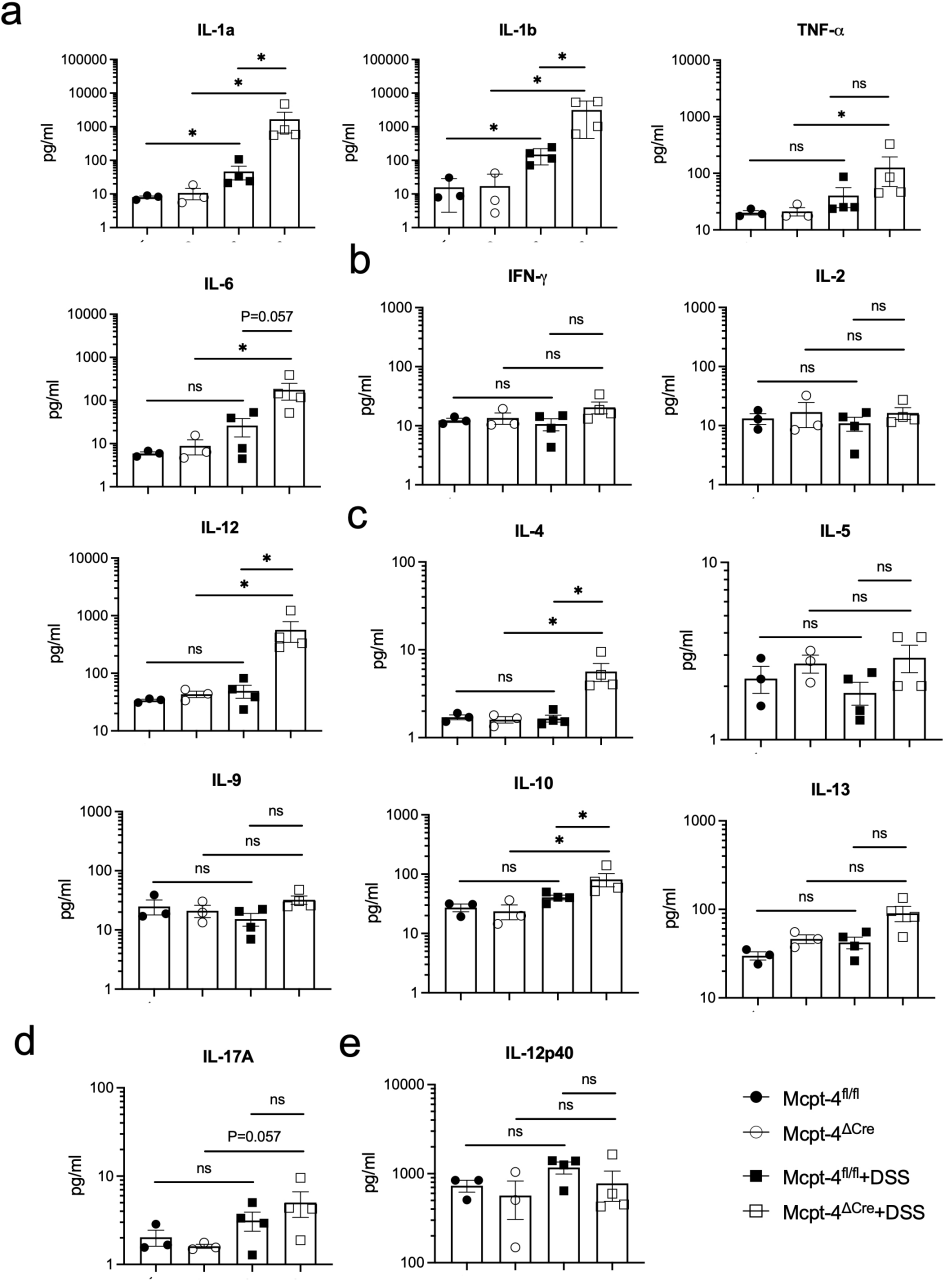
The colitis mice lacking the chymase Mcpt-4 gene have significantly higher levels of cytokines. The production of proinflammatory cytokines IL-1a, IL-1b, TNF-a, and IL-6 **(a)**; Th1 cytokines IFN-*γ*, IL-2, and IL-12 **(b)**; Th2 cytokines IL-4, IL-5, IL-9, IL-10, and IL-13 **(c)**; Th17 cytokine IL-17A **(d)**; and IL-12P40 **(e)**; the subunit of both IL-12 and IL-23 in colon tissue of mice (*n* ≧ 3) for each group was measured by Luminex multiplex assay. Data are shown as mean ± SE, and statistical analysis was conducted by Welch’s *t*-test with significant difference presented as *p < 0.05, ns, no significance. Note that there is no significance between groups Mcpt-4^fl/fl^ and Mcpt-4^ΔCre^.

To further confirm the effect of mMCP-4 on colitis, we next examined the production of the associated chemokines KC/CXCL1, MCP-1/CCL2, MIP-1a/CCL3, MIP-1b/CCL4, RANTES/CCL5, CCL11, G-CSF, and GM-CSF; proinflammatory cytokines IL-1a, IL-1b, TNF-a, and IL-6; Th1 cytokines IFN-*γ*, IL-2, and IL-12; Th2 cytokines IL-4, IL-5, IL-9, IL-10, and IL-13; Th17 cytokine IL-17A; and IL-12p40 in colon tissue of experimental mice. Note, importantly, that unchallenged Mcpt-4^fl/fl^ and Mcpt-4^ΔCre^ mice had similar levels of the chemokines and cytokines (**Figures 4**, **5**), suggesting that the Mcpt4 depletion did not alter the chemokine/cytokine expression in the colon tissue in steady state. However, and in contrast, the DSS-induced colitis caused significantly increased levels of CXCL1, CCL2, CCL3, CCL11, G-CSF, and GM-CSF in Mcpt-4^ΔCre^ mice as compared to Mcpt-4^fl/fl^ mice (**Figures 4a–c, f–h**). In addition, the colitis induced a significant increase in the chemokines CCL4 and CCL5, although without significant differences between the Mcpt-4^fl/fl^ and Mcpt-4^ΔCre^ colitis mice (**Figures 4d, e**).

Regarding pro-inflammatory cytokines, colitis caused a significant increase of IL-1α and IL-1β in both Mcpt-4^fl/fl^ and Mcpt-4^ΔCre^ mice, with Mcpt-4 deficiency promoting higher production of IL-1α and IL-1β (**Figure 5a**). TNF-α and IL-6 levels were significantly higher in Mcpt-4^ΔCre^ colitis mice compared to control Mcpt-4^ΔCre^ mice. Although no statistical significance was detected, a minor increase in these two cytokines was noted between Mcpt-4^fl/fl^ and Mcpt-4^ΔCre^ colitis mice (**Figure 5a**). In **Figures 5b, c**, colitis induced significant differences in IL-4, IL-10, and IL-12 levels in Mcpt-4^ΔCre^ mice, where no significant changes were observed in Mcpt-4^fl/fl^ colitis mice. Significant differences in chemokine levels were detected between Mcpt-4^fl/fl^ and Mcpt-4^ΔCre^ colitis mice (**Figure 5b**). Finally, no significant differences were observed for IFN-γ, IL-2, IL-5, IL-9, IL-13, IL-17A, and IL-12p40 (**Figures 5b–e**). Collectively, these findings strongly suggest that Mcpt-4 deficiency exacerbates colitis-associated inflammation.

### 3.4 The Mcpt-4 deficiency shifted the colonic microbiota of colitis mice

To explore how the Mcpt-4 deficiency potentially aggravates colitis, we next addressed the colonic microbiota. 16S rRNA genes were sequenced within the microbial genomic DNA extracted from colon contents. We initially compared the baseline microbiota composition between Mcpt-4^fl/fl^ and Mcpt-4^ΔCre^ mice. α-diversity metrics represented by the ace, sobs, chao, coverage, Simpson, or Shannon index were calculated to assess microbial richness and diversity in Mcpt-4^fl/fl^ and Mcpt-4^ΔCre^ mice. Although Mcpt-4^ΔCre^ mice exhibited slightly higher mean richness, none of these differences were statistically significant (**Supplementary Figure 3a**). β-diversity using PCoA based on OTU-level data revealed no significance in overall microbial community structure between the two genotypes (**Supplementary Figure 3b**). The Venn diagram illustrated a total of 86 OTUs shared between the two genotypes, with 5 unique to Mcpt-4^fl/fl^ mice and 9 unique to Mcpt-4^ΔCre^ mice (**Supplementary Figure 4a**). LEfSe results illustrate bacterial taxa that are significantly enriched in each genotype, where 26 taxa were enriched in Mcpt-4^ΔCre^ mice and 6 taxa enriched in Mcpt-4^fl/fl^ mice (**Supplementary Figure 4b**). Community barplot analysis identified the relative abundances of bacterial genera in Mcpt-4^fl/fl^ and Mcpt-4^ΔCre^ groups, and the overall community profiles appeared similar (**Supplementary Figure 4c**).

Microbiota in DSS-induced colitis Mcpt-4^fl/fl^ + DSS and Mcpt-4^ΔCre^ + DSS groups was compared and analyzed. In **Supplementary Figure 5a**, ace index values demonstrated that microbial richness was significantly reduced in Mcpt-4^fl/fl^ + DSS mice, while Mcpt-4^ΔCre^ + DSS mice maintained significantly higher richness (*p* = 0.0031), indicating greater microbial resilience. PCoA showed distinct clustering between these groups (*R*² = 0.2507, *p* = 0.044), indicating a genotype-dependent divergence in microbiota structure under DSS challenge (**Supplementary Figure 5b**). **Supplementary Figure 6a** shows a Venn diagram with 79 shared OTUs, and 7 and 14 OTUs unique to Mcpt-4^fl/fl^ + DSS and Mcpt-4^ΔCre^ + DSS, respectively. LEfSe results revealed significant enrichment of multiple taxa in Mcpt-4^ΔCre^ + DSS mice, including *Akkermansia, Turicibacter*, and *Clostridia*, while Mcpt-4^fl/fl^ + DSS mice were enriched in *Bacteroides* and *Klebsiella* (**Supplementary Figure 6b**). Community barplot analysis showed altered microbial profiles consistent with these LEfSe results (**Supplementary Figure 6c**).

**Figure 6:**
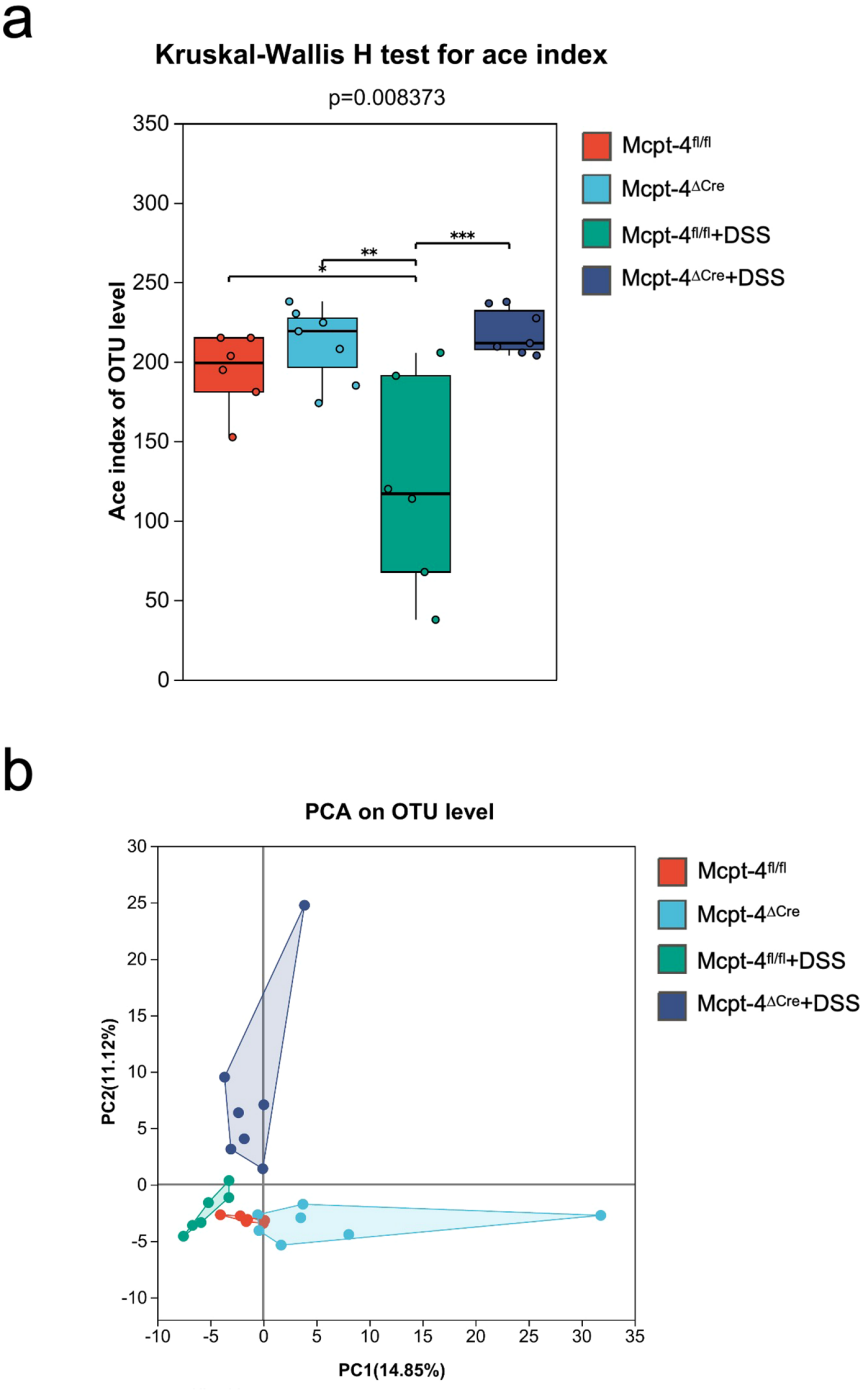
The Mcpt-4 deficiency alters the composition of gut microbiota in colitis mice. Colon samples were collected from mice (*n* ≧ 6 in each group) with colitis. 16S rRNA genes were sequenced with the microbial genomic DNA extracted from colon contents. α-diversity comparison among experimental groups using Kruskal–Wallis *H* test was represented by the Ace index **(a)**. β-diversity represented by principal component analysis (PCA) on OTU level was analyzed and the variance was presented by the principal coordinate (PC) **(b)**. Significant difference are presented as *p < 0.05, **p < 0.01 and ***p < 0.001.

**Figure 7:**
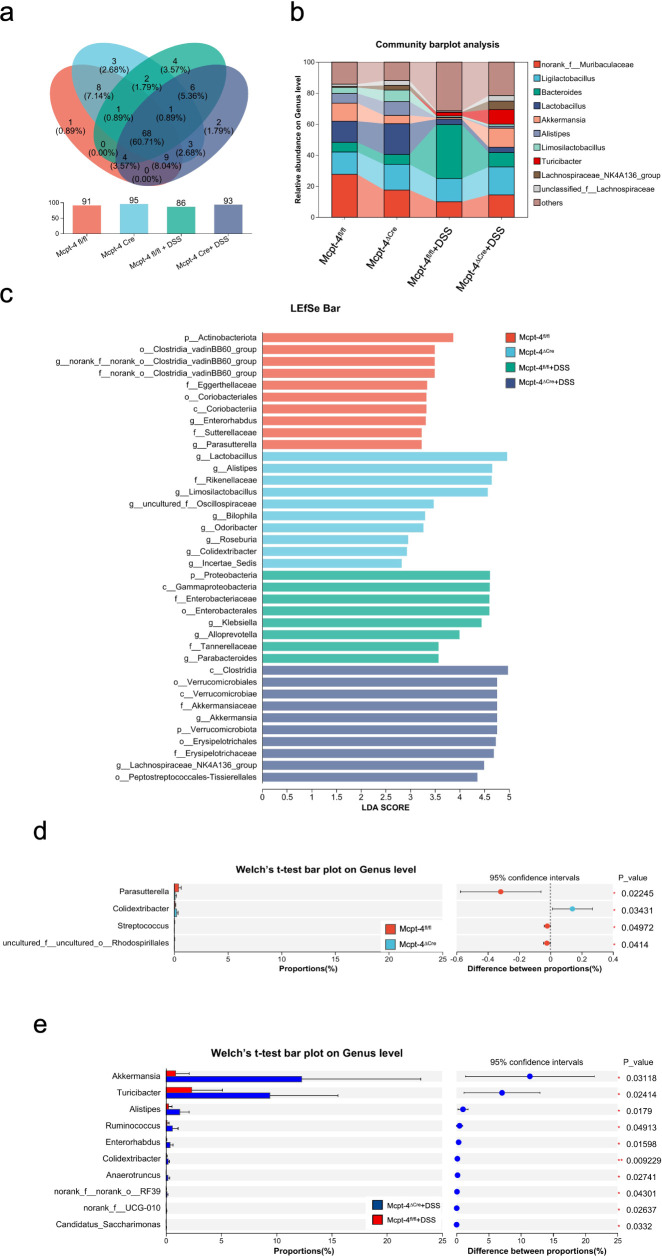
The Mcpt-4 deficiency distorts the colonic microbiota of colitis mice. Venn diagram showing shared and unique operational taxonomic units (OTUs) among groups **(a)**. Community barplot analysis at the genus level displaying the relative average abundance of major bacterial taxa across groups **(b)**. Linear discriminant analysis effect size (LEfSe) analysis identifying differentially enriched taxa among groups, with a threshold LDA score >2.0 and *p* < 0.05 **(c)**. Welch’s *t*-test barplot comparing genus-level abundances between Mcpt-4^fl/fl^ and Mcpt-4^ΔCre^ groups, and Mcpt-4^fl/fl^ + DSS and Mcpt-4^ΔCre^ + DSS groups. Only genera showing statistically significant differences (*p* < 0.05) are shown **(d, e)**.

An integrated microbiota analysis was performed to evaluate differences across all experimental groups. α-diversity was assessed using the ace index, and the Kruskal–Wallis *H* test revealed significant differences across groups (*p* = 0.008373). Mcpt-4^fl/fl^ and Mcpt-4^ΔCre^ mice showed comparable richness at baseline. Following DSS treatment, microbial richness was significantly reduced in Mcpt-4^fl/fl^ + DSS mice (*p* < 0.01 vs. Mcpt-4^fl/fl^), whereas this reduction was not observed in Mcpt-4^ΔCre^ + DSS mice. Notably, Mcpt-4^ΔCre^ + DSS mice retained significantly higher ace indices compared to Mcpt-4^fl/fl^ + DSS mice (**Figure 6a**). PCA based on OTU-level data assessing β-diversity across all groups revealed a distinct clustering pattern among the four experimental groups, with the Mcpt-4^ΔCre^ + DSS group forming a separate cluster from the others, particularly Mcpt-4^fl/fl^ + DSS. The first two principal coordinates (PC1 and PC2) explained 14.85% and 11.72% of the variance, respectively (**Figure 6b**). The Venn diagram showed that a core set of 60 OTUs was shared across all groups, with each group also possessing a small number of unique OTUs (**Figure 7a**). Community barplot analysis showed that Mcpt-4^ΔCre^ + DSS mice exhibited altered microbial composition, including visible changes in the abundance of genera such as *Akkermansia, Turicibacter*, and *Alistipes* (**Figure 7b**). LEfSe identified differentially abundant taxa among groups. There were 10 enriched in Mcpt-4^fl/fl^ mice, 10 in Mcpt-4^ΔCre^ mice, 8 in Mcpt-4^fl/fl^ + DSS mice, and 10 in Mcpt-4^ΔCre^ + DSS mice (**Figure 7c**). Further analysis using Welch’s *t*-test revealed significant differences between Mcpt-4^fl/fl^ and Mcpt-4^ΔCre^ mice without DSS treatment in the abundance of genera *Parasutterella, Colidextribacter, Streptococcus*, and *uncultured Rhodospirillales*, of which all had low abundance (**Figure 7d**). Comparison between DSS-treated Mcpt-4^fl/fl^ and Mcpt-4^ΔCre^ mice identified multiple genera *norank_f:norank_o:RF39*, *Akkermansia, Turicibacter, Alistipes, Ruminococcus*, *Enterorhabdus*, *Colidextribacter, Anaerotruncus, norank_f:UCG-010*, and *Candidatus_Saccharimonas* that were significantly enriched in Mcpt-4^ΔCre^ + DSS mice (**Figure 7e**).

This multi-level microbiota analysis reveals that while Mcpt-4 deficiency exerts only subtle effects on microbiota at baseline, it significantly reshapes the gut microbial landscape during colitis, indicating a critical role for mouse chymase MCP-4 in modulating host–microbiota interactions and maintaining gut microbial homeostasis under colitis.

### 3.5 The lack of Mcpt-4 distorted the colonic metabolism of colitis mice

**Figure 8:**
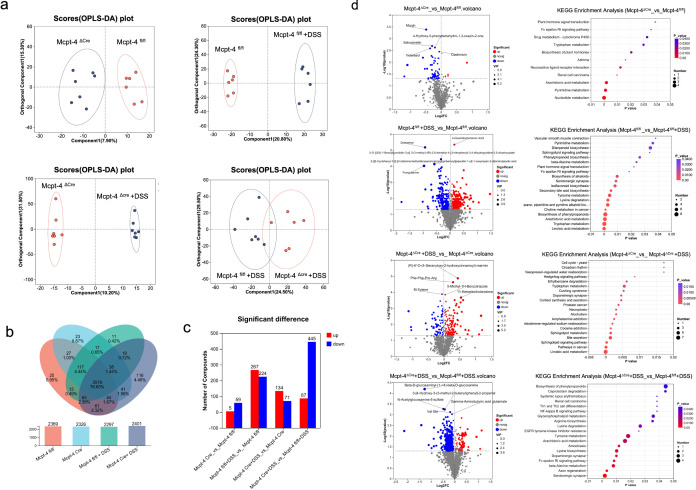
The lack of Mcpt-4 disrupts the colonic metabolism of colitis mice. OPLS-DA analysis score plots showing comparisons of the metabolome profiles between Mcpt-4^fl/fl^ and Mcpt-4^ΔCre^, Mcpt-4^fl/fl^ and Mcpt-4^fl/fl^ +DSS, Mcpt-4^ΔCre^ and Mcpt-4^ΔCre^ +DSS, and Mcpt-4^fl/fl^ +DSS and Mcpt-4^ΔCre^ +DSS **(a)**. The identified metabolites were counted and the overlap among groups was shown with the Venn diagram **(b)**. Barplots showing the number of significantly upregulated and downregulated metabolites identified in each pairwise group comparison (VIP > 1, *p* < 0.05) **(c)**. Volcano plots illustrating significantly altered metabolites between groups; the top five upregulated and downregulated metabolites are labeled (**d**, left panel). KEGG pathway enrichment bubble plot showing the top 20 significantly enriched metabolic pathways (*p* < 0.05) among the four groups, based on differentially abundant metabolites (**d**, right panel).

The metabolism in colon is also highly correlated to the inflammatory level during colitis (Yuan et al., 2021). Next, we performed metabolomics using the colonic content of experimental mice. A comprehensive metabolomic analysis compared Mcpt-4^fl/fl^ and Mcpt-4^ΔCre^ mice under both basal and DSS-induced colitis conditions. **Figure 8a** presents OPLS-DA plots that demonstrate distinct clustering of metabolic signatures among all experimental groups. Specifically, the comparison between Mcpt-4^fl/fl^ and Mcpt-4^ΔCre^ mice under basal conditions revealed well-separated metabolic profiles, highlighting genotype-dependent differences. Similarly, comparisons between Mcpt-4^fl/fl^ and Mcpt-4^fl/fl^ + DSS, and between Mcpt-4^ΔCre^ and Mcpt-4^ΔCre^ + DSS demonstrated the metabolic impact of DSS-induced colitis within each genotype. Notably, the comparison between Mcpt-4^fl/fl^ + DSS and Mcpt-4^ΔCre^ + DSS comparisons emphasized the combined effects of Mcpt-4 deficiency and colonic inflammation on metabolic alterations. The Venn diagram and bar chart summarized the number and overlap of detected metabolites across all groups (**Figure 8b**). The identified metabolites in total for each group were 2,369 in group Mcpt-4^fl/fl^, 2,326 in group Mcpt-4^ΔCre^, 2,297 in group Mcpt-4^fl/fl^ +DSS, and 2,401 in group Mcpt-4^ΔCre^ +DSS, of which 2,019 metabolites (76.56% of the total) were shared among all four groups, while a modest number of unique metabolites were identified in each condition (e.g., 25 in Mcpt-4^fl/fl^, 27 in Mcpt-4^ΔCre^, 13 in Mcpt-4^fl/fl^ + DSS, and 19 in Mcpt-4^ΔCre^ + DSS) (**Figure 8b**). **Figure 8c** displays the number of significantly altered compounds between groups. The most dramatic difference was observed between Mcpt-4^ΔCre^ and Mcpt-4^fl/fl^ colitis mice, where 445 metabolites were significantly downregulated and 87 were upregulated in the Mcpt-4-deficient group. Between Mcpt-4^fl/fl^ and Mcpt-4^fl/fl^ + DSS mice, 267 metabolites were downregulated and 224 were upregulated.

**Figure 8d** presents the integrating KEGG pathway enrichment to reveal distinct metabolic alterations. The corresponding KEGG pathway enrichment analysis revealed that Mcpt-4^ΔCre^ mice had significant regulation in nucleotide metabolism, pyrimidine metabolism, tryptophan metabolism, and cytochrome P450-mediated xenobiotic metabolism compared to Mcpt-4^fl/fl^. In DSS-treated Mcpt-4^fl/fl^ mice, pathways involved in sphingolipid signaling, phenylpropanoid biosynthesis, and inflammatory lipid mediators were significantly enriched. In contrast, DSS-treated Mcpt-4^ΔCre^ mice exhibited broader metabolic disruption, with enrichment in pathways including cortisol synthesis, dopaminergic synapse signaling, hedgehog signaling, and the cell cycle. The most profound alterations were observed when comparing DSS-treated Mcpt-4^ΔCre^ to DSS-treated Mcpt-4^fl/fl^ mice, with enrichment in serotonergic synapse, axon regeneration, β-alanine metabolism, Fc epsilon RI signaling pathway, dopaminergic synapse, lysine biosynthesis, amoebiasis, arachidonic acid metabolism, tyrosine metabolism, EGFR tyrosine kinase inhibitor resistance, lysine degradation, glycerophospholipid metabolism, Th1 and Th2 cell differentiation, renal cell carcinoma, systemic lupus erythematosus, NF-kappa B signaling pathway, caprolactam degradation, and biosynthesis of phenylpropanoids.

Together, these indicate that Mcpt-4 plays a critical role in maintaining metabolic homeostasis and modulating metabolic responses to DSS. The altered metabolic profiles observed in Mcpt-4^ΔCre^ mice, especially under colitis conditions, highlight how Mcpt-4 impacts colonic metabolic pathways, potentially influencing colitis severity via the regulation of metabolites.

### 3.6 Correlation of colonic cytokines with microbiota and metabolites

Next, we conducted a comprehensive analysis of the correlations between cytokine levels and both microbial taxa and metabolites in DSS-induced colitis to evaluate the key microbe–host and metabolite–host interactions modulated by Mcpt-4 deficiency. In **Figure 1a**, the Spearman correlation heatmap depicts associations between the detected cytokines and gut microbial genera (top 50). Notably, several bacterial taxa, such as *Desulfovibrio, GCA-900066575, Unclassified_f:Oscillospiraceae, Clostridium_sensu_stricto_1, Romboutsia*, *Eubacterium_siraeum_group*, and *Turicibacter*, displayed significant positive correlations with most of detected cytokines (red arrows and shading in **Figure 1a**), indicating the potential contributions of these genera to colonic inflammation in Mcpt-4^ΔCre^ DSS mice. Conversely, genera such as *Lachnospiraceae_UCG-001*, *Clostridium_Innocuum_Group*, *Lactobacillus*, *Odoribacter, Alistipes*, and *Limosilactobacillus* exhibited negative correlations with those determined cytokines (blue arrows and shading in **Figure 1a**), suggesting potential anti-inflammatory roles. **Figure 1b** presents a Spearman correlation heatmap analyzing associations between individual metabolites and cytokine levels. Notably, nearly all detected cytokines, except IL-12p40, RANTES/CCL5, and MIP-1beta, exhibited strong positive correlations with three specific metabolites: Sorbitan palmitate, Gamma-glutamylvaline, and Sorbitan stearate. Conversely, a group of 13 metabolites, namely, Val-Leu, Ile-Leu, leucyl-leucine, Ser-Leu, Leu-Ser, Ile-Val, Val-Ile, gamma-glutamylleucine, Glu-Val, ecgonine methyl ester, Leu-Thr, Val-Glu, and Ile-Glu, were consistently and strongly negatively correlated with most cytokines. These findings suggest that the absence of Mcpt-4 leads to metabolic shifts that are tightly linked to elevated inflammatory cytokine expression, highlighting a potential mechanistic role for Mcpt-4 in modulating metabolite–immune interactions during colitis.

## 4 Discussion

UC is a chronic inflammatory disease characterized by recurrent episodes of diarrhea and abdominal pain, which are manifestations of underlying colonic inflammation. Previous studies have documented elevated chymase levels in patients with UC, and inhibition of chymase has been shown to mitigate the severity of inflammatory bowel disease (Yang and Merlin, 2024). To elucidate the role of chymase in the pathogenesis of UC, we developed a murine model of colitis through induction with DSS. Given that murine mMCP-4 shares functional and structural homology with human chymase, we conducted further investigations using Mcpt-4-deficient (Mcpt-4^ΔCre^) mice to determine the specific involvement of mMCP-4 in the pathophysiology of DSS-induced colitis.

DSS-induced colitis in mice is a well-established experimental model, characterized by significant weight loss, reduced colon length, and pronounced immune cell infiltration leading to extensive tissue damage within the colon (Yang and Merlin, 2024). Our study demonstrates that the absence of Mcpt-4 exacerbates the pathological manifestations of DSS-induced colitis in mice. Specifically, Mcpt-4-deficient mice exhibited markedly intensified colonic inflammation compared to their control counterparts, as evidenced by more severe tissue destruction and enhanced infiltration of inflammatory cells. The deficiency of Mcpt-4 appears to compromise the protective mechanisms typically activated during colitis, resulting in increased susceptibility to DSS-induced colonic injury. This suggests a protective role for mMCP-4 in maintaining colonic homeostasis and modulating inflammatory responses during colitis. Interestingly, this effect contrasts with previous findings where colitis severity was mitigated by treatment with a chymase inhibitor (Liu et al., 2016). Considering that mouse mast cells express five distinct chymases, the role of mMCP-4 in colitis may be partially compensated or modulated by other chymases, thereby restricting its functional impact in colitis.

Pro-inflammatory cytokines are critically involved in the pathogenesis and progression of UC (Nakase et al., 2022). These signaling molecules, produced by various cell types, orchestrate the inflammatory response, thereby contributing to mucosal injury. In our study, we measured the levels of chemokines and pro-inflammatory cytokines highly associated with UC, of which chemokines CXCL1, CCL2, CCL3, CCL4, CCL5, and CCL11 were reported to promote UC inflammation (Yu et al., 2016; Trivedi and Adams, 2018; Polosukhina et al., 2021; Zhang et al., 2023). In our work, the defect of Mcpt-4 gene led to colitis mice producing higher levels of chemokines especially CXCL1, CCL2, CCL3, and CCL11. G-CSF and GM-CSF are two important cytokines in promoting cell proliferation and differentiation. Both were evidenced to be protective in DSS-induced colitis mice (Xu et al., 2008; Meshkibaf et al., 2016). Here, the colitis induced higher levels of these two cytokines in Mcpt-4^ΔCre^ mice but not in Mcpt-4^fl/fl^ mice. This unclear finding needs to be further studied and clarified.

TNF-α and IL-6 are two pro-inflammatory cytokines in UC that cause the exacerbation of inflammatory responses. Thus, targeting them with specific inhibitors or antibodies has been shown to ameliorate UC symptoms. For instance, inhibitors and antibodies against tumor necrosis factor-alpha (TNF-α) have proven effective in reducing inflammation and inducing remission in UC patients (Straatmijer et al., 2023). Additionally, inhibitors of IL-6 are currently under investigation for their therapeutic potential in UC. In our work, the levels of TNF-α and IL-6 did not statistically significantly increase in Mcpt-4^fl/fl^ colitis mice compared with that in control Mcpt-4^fl/fl^ mice, but there was a minor increase. However, the lack of Mcpt-4 could induce significant increase of TNF-α and IL-6 in colitis mice. Although no statistical significance was detected, a minor increase was noted between Mcpt-4^fl/fl^ and Mcpt-4^ΔCre^ colitis mice.

UC has been characterized with a pending mixture of Th1, Th2, and Th17 immune responses linked to the predominant phase of UC (Nakase et al., 2022). Here, our data suggest that the deficiency of Mcpt-4 triggered a stronger Th2 response in colitis as manifested by increased levels of IL-4, IL-5, IL-10, and IL-13, whereas IL-10 is also generally known as an anti-inflammatory cytokine (Fan et al., 2021). Mice with IL-10 deficiency can obtain spontaneous colitis (Jia et al., 2022). On this aspect, they play protective roles in UC. Here, we found that Mcpt-4-deficient colitis mice had significantly higher levels of IL-10 compared to Mcpt-4^ΔCre^ colitis or Mcpt-4^fl/fl^ mice. Given that mice were allowed a 2-day water recovery period after 7 days of DSS exposure, this elevation in IL-10 may reflect the initiation of a compensatory anti-inflammatory response during the early resolution phase of colitis. Thus, the observed IL-10 elevation in Mcpt-4-deficient colitis mice may indicate an endogenous attempt to mitigate inflammation that is otherwise exacerbated in the absence of Mcpt-4. IL-12, IL-1a, and IL-1b are refined as Th1 cytokines that mainly exert a pro-inflammatory function in UC (Fan et al., 2021). In the present study, Mcpt-4 deficiency could lead to a significant increase in colitis mice, which can clearly account for why the colonic inflammation was exacerbated in Mcpt-4^ΔCre^ colitis mice.

Previous studies have indicated that mMCP-4 can modulate cytokine expression in the gut during *Giardia* infection (Li et al., 2020), suggesting a potential role in mitigating colitis through the regulation of colonic cytokines. Our data further support this hypothesis, showing that the absence of Mcpt-4 leads to increased production of inflammatory cytokines in mice with colitis. The ability of mMCP-4 to regulate cytokine production implies that modulating its function could be a viable strategy for controlling the inflammatory response in colitis. This approach may offer novel therapeutic avenues, potentially improving disease management and patient outcomes in UC.

The gut microbiota, consisting of trillions of microorganisms residing in the intestinal tract, plays a crucial role in maintaining intestinal homeostasis and immune function. Disruption of the gut microbiota, often referred to as dysbiosis, has been linked to the pathogenesis of UC (Yuan et al., 2021). The metabolic alterations in the host can both influence and be influenced by the state of gut microbiota, influencing the severity of inflammation and the overall health of the colonic mucosa (Yuan et al., 2021). An interesting finding showed that Mcpt-4 deficiency could impair the intestinal homeostasis and barrier function in mice (Groschwitz et al., 2009). In our study, Mcpt-4 deficiency alone did not significantly alter levels of the measured cytokines under basal conditions. Based on these findings, we hypothesized that Mcpt-4 may influence intestinal homeostasis not directly through cytokine modulation, but rather via alterations in gut microbiota composition or metabolic pathways. To investigate this, we compared the microbiota and colonic metabolite profiles between Mcpt-4^ΔCre^ and Mcpt-4^fl/fl^ mice without DSS treatment under steady-state conditions. While only modest differences in microbiota composition were observed, these were accompanied by more noticeable alterations in metabolite profiles, suggesting that mMCP-4 may contribute to gut health and disease susceptibility such as colitis, through its impact on intestinal metabolism rather than immune signaling alone.

To address the underlying mechanism on the Mcpt-4 deficiency promoting colitis, we further analyzed the microbiota and metabolism using gut contents. In our work, an imbalance in the composition of bacterial communities was more pronounced in Mcpt-4-deficient colitis mice. In comparison, between Mcpt-4^fl/fl^ and Mcpt-4^ΔCre^ colitis mice, the deficiency of Mcpt-4 predominantly supplied the significance of bacteria including the predominant genera *Akkermansia* and *Turicibacter*. Together with *Alistipes, Ruminococcus, Enterorhabdus, Colidextribacter*, and *Anaerotruncus*, the roles of both genera in colitis were found to be controversial because of their double-faced functions on the development of colitis. For example, *Akkermansia* is generally considered as a probiotic, and it has been shown that the administration of *Akkermansia muciniphila* ameliorated DSS-induced experimental colitis in mice (Bian et al., 2019), whereas according to Håkansson’s team, colonic samples of DSS-induced colitis mice showed higher *Akkermansia* abundance than their counterparts in the control group (Zheng et al., 2022). In a recent article, salidroside was found to alleviate colitis by decreasing *Turicibacter* and *Alistipes* (Liu et al., 2023), indicating their harmful role in colitis. In our work, the deficiency of Mcpt-4 induced the significant increase in abundance of the potential harmful bacteria *Akkermansia, Turicibacter*, and *Alistipes*. These findings reveal that the absence of Mcpt-4 may affect the microbiota through direct interactions with microbial populations or indirectly by altering the immune environment within the gut. These results uncover that mMCP-4 might influence the microbiota by promoting a healthy microbial environment.

Mcpt-4 is known to influence local tissue remodeling and vascular permeability (Pejler, 2020), and our findings suggest that these effects may extend to modulating the production, transformation, or bioavailability of metabolites that participate in immune signaling and epithelial function. Several differentially abundant metabolites identified in Mcpt-4-deficient mice are known to be associated with immune modulation, epithelial barrier integrity, or microbial metabolism. These changes may sensitize the mucosa to inflammatory insults or alter immune tone, thus predisposing Mcpt-4^ΔCre^ mice to more severe colitis upon DSS treatment. For example, in the top 20 enriched KEGG pathways, the NF-Kappa B signaling pathway and Th1 and Th2 cell differentiation were extensively reported in studies on UC etiology. The inhibition of the NF-Kappa B signaling pathway-induced inflammatory responses by a potential medicine could ameliorate UC (Laurindo et al., 2023). The shift of Th1 and Th2 cells affected the development of UC inflammation (Gomez-Bris et al., 2023). These findings reveal that the disruption of colonic metabolism in Mcpt-4-deficient mice underscores the interconnectedness of metabolism and inflammation in DSS-induced colitis. Mcpt-4 deficiency leads to metabolic reprogramming in the gut that may not be fully captured by microbial taxonomic shifts alone. Instead, the metabolic environment created by the absence of Mcpt-4 may foster a pro-inflammatory state or reduce the threshold for mucosal injury, contributing to the exacerbated colitis observed in Mcpt-4^ΔCre^ mice.

The intricate correlation of cytokine production with microbiota composition and metabolic profiles in Mcpt-4-deficient mice highlights the complexity of colitis pathogenesis. In colitis, a similar triad of distorted cytokine signaling, dysbiosis, and metabolic imbalance drives the chronic inflammatory state. Elevated pro-inflammatory cytokines can alter the microbiota, leading to dysbiosis, which, in turn, affects metabolic pathways, creating a cycle that perpetuates inflammation. Understanding these interactions in the context of Mcpt-4 deficiency provides a comprehensive picture of colitis pathology and identifies multiple points for potential intervention. Interestingly, among the bacteria genera shown in **Figure 8**, only *Alistipes* had a significant correlation with most of the detected cytokines and metabolites (**Supplementary Figure 7**). It would be promising to further investigate the role of *Alistipes* in Mcpt-4-deficient mice with exacerbated colitis.

**Figure 9:**
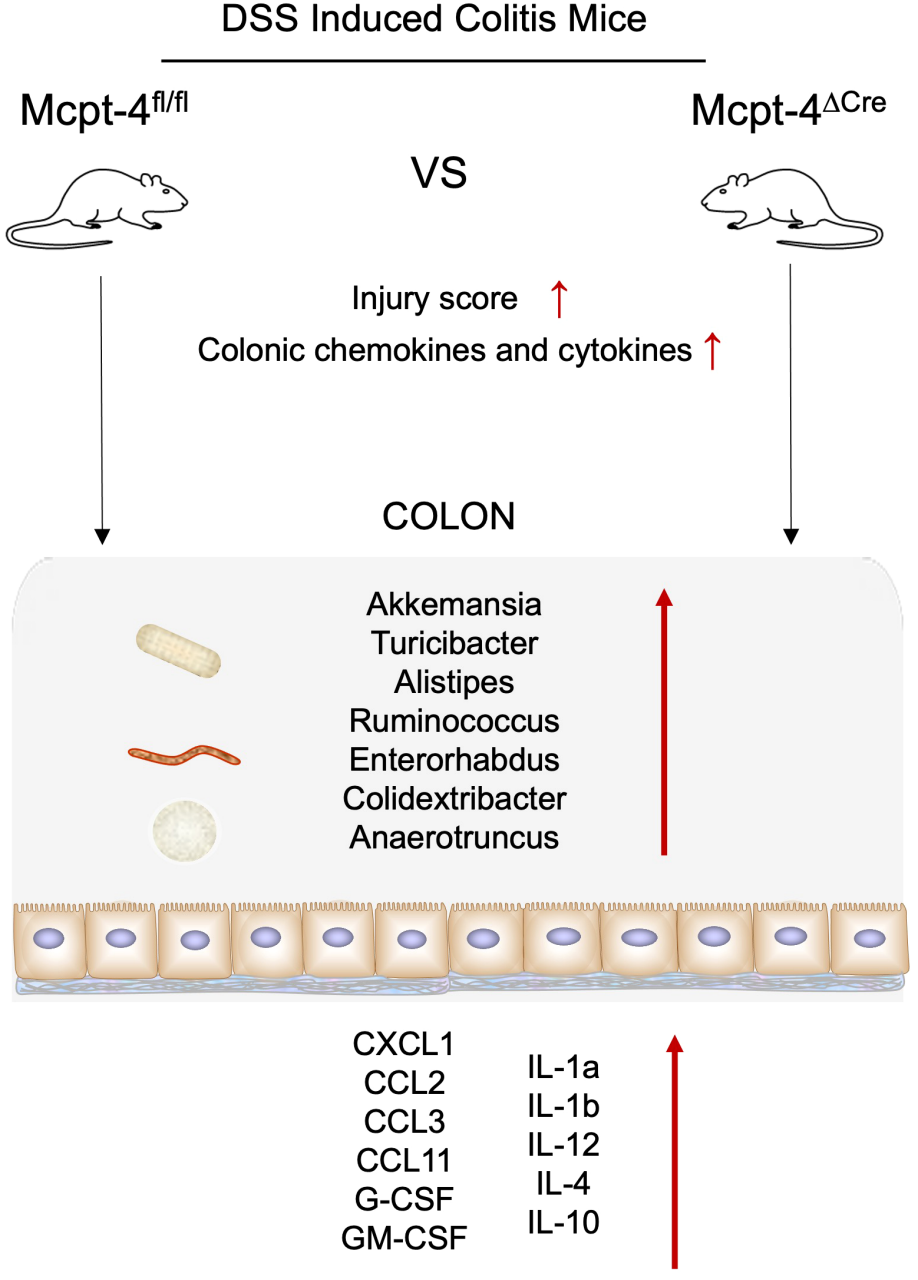
Schematic image. The absence of Mcpt-4 leads to a more severe inflammatory response, characterized by reduced colon length, injury score, increased pro-inflammatory chemokine and cytokine production, dysbiosis, and disrupted metabolic profiles.

In summary, our study underscores the crucial role of mMCP-4 in modulating colonic inflammation, microbiota, and metabolism during DSS-induced colitis. The absence of Mcpt-4 leads to a more severe inflammatory response, characterized by increased inflammatory cytokine production, dysbiosis, and disrupted metabolic profiles (**Figure 9**). These findings highlight the importance of mMCP-4 in maintaining intestinal homeostasis and suggest that therapeutic strategies aimed at modulating mMCP-4 function might be beneficial in managing colitis and potentially other inflammatory bowel diseases. Further research is needed to elucidate the precise mechanisms by which the chymase mMCP-4 regulates these processes and to explore its potential as a therapeutic target.

## Data Availability

The datasets presented in this study are deposited in online repositories. The microbiota sequencing data are available in the NCBI Sequence Read Archive (SRA) under accession number PRJNA1150579. The metabolomics data are available on Figshare at the following link: https://figshare.com/s/693809d934f451ac0b0e. (DOI: 10.6084/m9.figshare.26039644). Now they are publicly released.

## Supplementary material

Supplementary Figure 1 Mcpt-4 gene knockout strategy **(a)**, Cre tool gene identification **(b)** and Mcpt-4 gene expression in the genotyped Cre mice **(c)**.

Supplementary Figure 2 Mice sex and numbers for each group in two independent experiments.

Supplementary Figure 3 A-diversity and β-diversity of gut microbiota in Mcpt-4^fl/fl^ and Mcpt-4^ΔCre^ mice. Colon samples were collected from Mcpt-4^fl/fl^ and Mcpt-4^ΔCre^ mice (n≧6 in each group). 16s rRNA genes were sequenced with the microbial genomic DNA extracted from colon contents. A-diversity comparison between Mcpt-4^fl/fl^ and Mcpt-4^ΔCre^ groups using student’s t-test was represented by the Ace, Sobs, Chao, Simpson coverage or Shannon index **(a)**. Principal Coordinates Analysis (PCoA) representing β-diversity on OTU level was analyzed **(b)**.

Supplementary Figure 4 The comparison of colonic microbiota of Mcpt-4^fl/fl^ and Mcpt-4^ΔCre^ mice. The microbiota data were further analyzed. The bacteria on genus level were counted between Mcpt-4^fl/fl^ and Mcpt-4^ΔCre^ groups **(a)**. The linear discriminant analysis of effect size was calculated **(b)**. The percent of community abundance of gut bacterial on genus levels was shown in **(c)**.

Supplementary Figure 5 The Mcpt-4 deficiency alters the composition of gut microbiota in colitis mice. Colon samples were collected from mice (n≧6 in each group) with colitis. 16s rRNA genes were sequenced with the microbial genomic DNA extracted from colon contents. A-diversity comparison between Mcpt-4^fl/fl^ +DSS and Mcpt-4^ΔCre^ +DSS groups using student’s t-test was represented by the Ace index **(a)**. Principal Component Analysis (PCA) representing β-diversity on OTU level was analyzed **(b)**.

Supplementary Figure 6 The Mcpt-4 deficiency distorts the colonic microbiota of colitis mice. The microbiota data were further analyzed. The bacteria on OTU level were counted between Mcpt-4^fl/fl^ +DSS and Mcpt-4^ΔCre^ +DSS groups **(a)**. The linear discriminant analysis of effect size was calculated **(b)**. The percent of community abundance of gut bacterial on genus levels was shown in **(c)**.

Supplementary Figure 7
*Alistipes* correlates with many cytokines and metabolites. Spearman correlation among colonic cytokines, microbiota (on genus level) and metabolite between Mcpt-4^fl/fl^ +DSS and Mcpt-4^ΔCre^ +DSS groups were analyzed. The red color indicates a positive correlation, while blue color indicates a negative correlation.
