# Supplementary figures and images for: The deficiency of chymase mast cell protease 4 exacerbates dextran sulfate sodium salt-induced colitis in mice and is associated with altered microbiota and metabolome profiles
Source: Front Cell Infect Microbiol. 2025 Jul 8;15:1481927. doi: 10.3389/fcimb.2025.1481927 (PMC12279878; doi:10.3389/fcimb.2025.1481927)

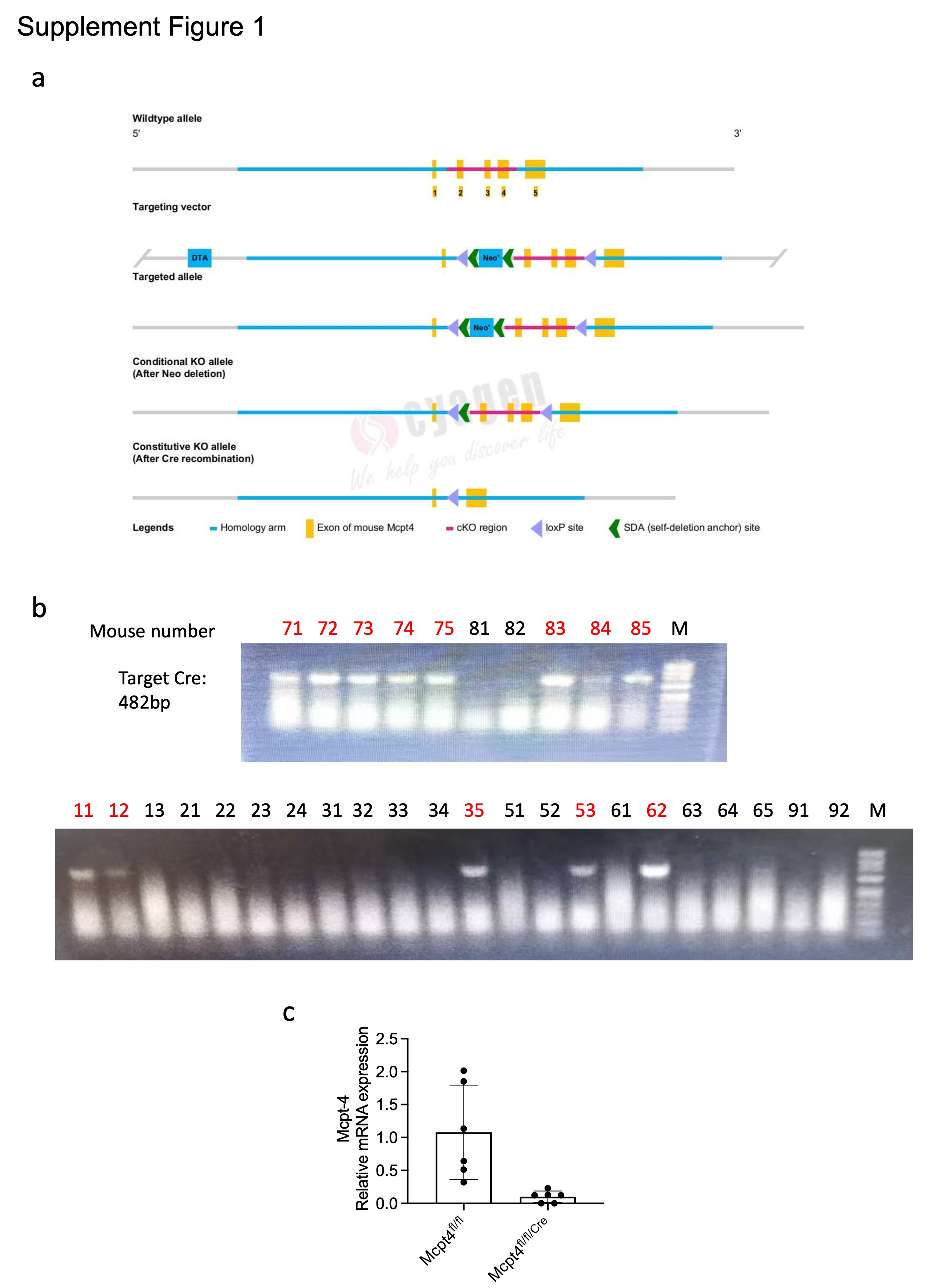

Supplement: Supplementary Figure 1 — Mcpt-4 gene knockout strategy (a), Cre tool gene identification (b) and Mcpt-4 gene expression in the genotyped Cre mice (c). [file Image1.jpg]

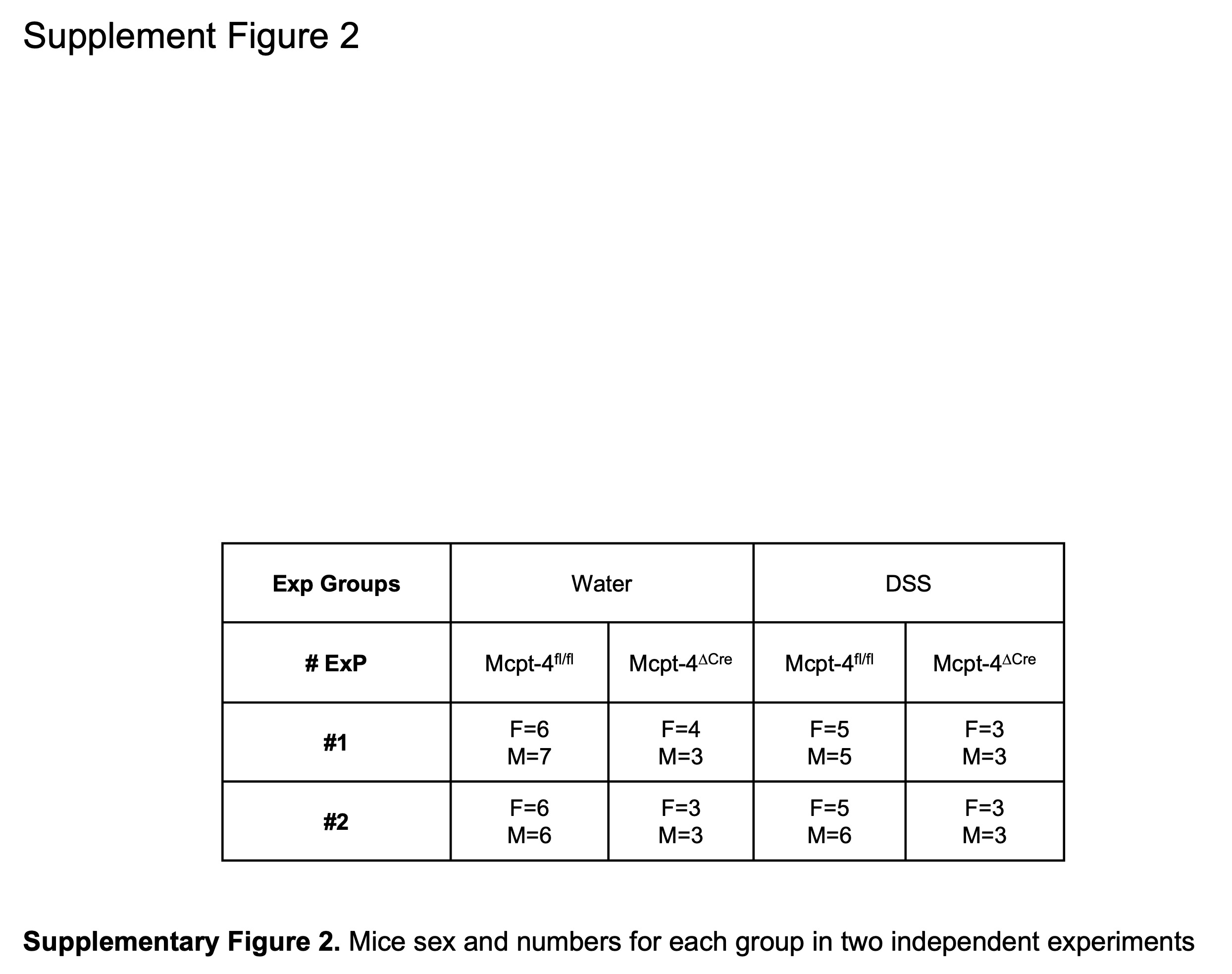

Supplement: Supplementary Figure 2 — Mice sex and numbers for each group in two independent experiments. [file Image2.jpg]

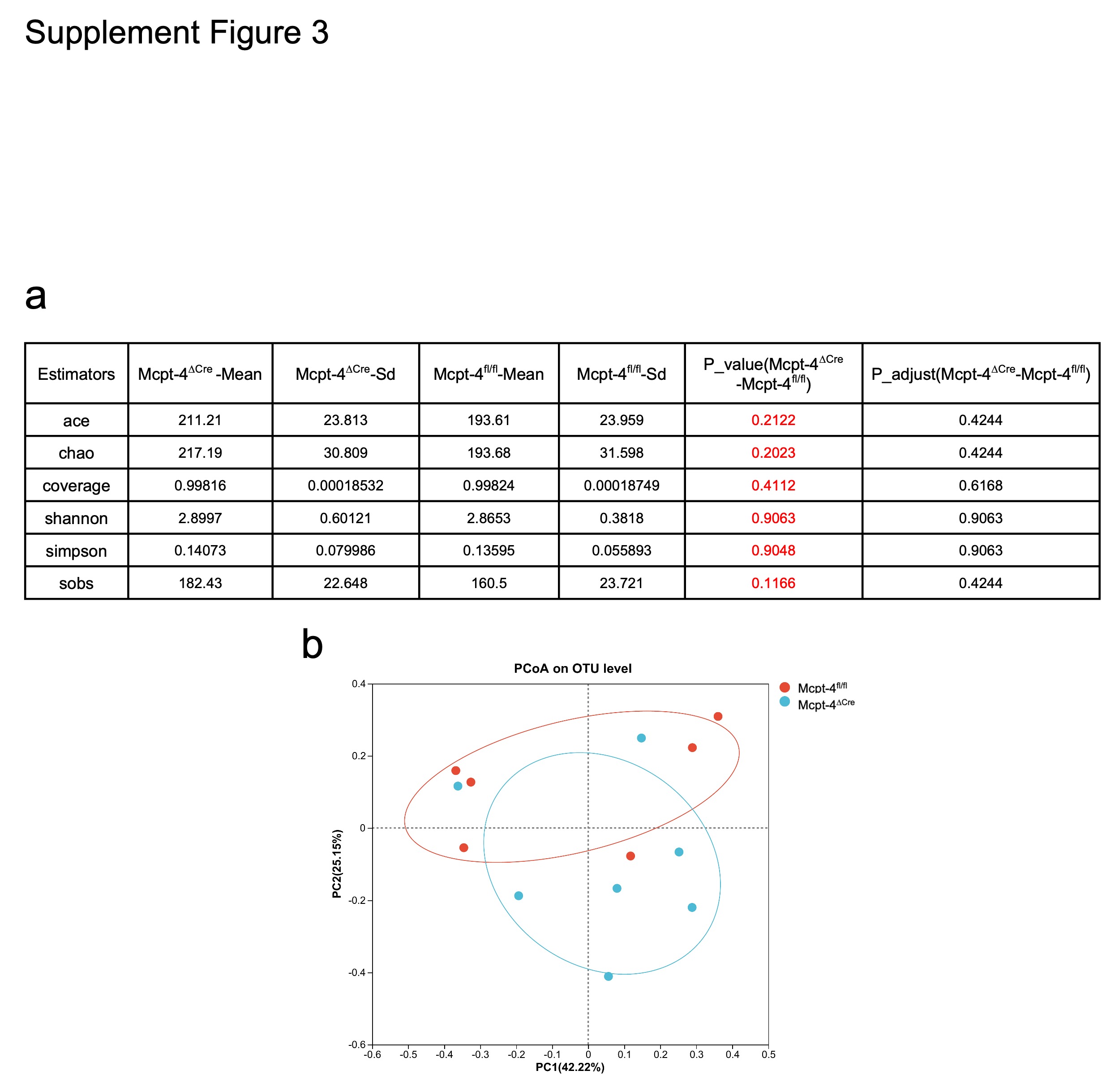

Supplement: Supplementary Figure 3 — A-diversity and β-diversity of gut microbiota in Mcpt-4fl/fl and Mcpt-4ΔCre mice. Colon samples were collected from Mcpt-4fl/fl and Mcpt-4ΔCre mice (n≧6 in each group). 16s rRNA genes were sequenced with the microbial genomic DNA extracted from colon contents. A-diversity comparison between Mcpt-4fl/fl and Mcpt-4ΔCre groups using student’s t-test was represented by the Ace, Sobs, Chao, Simpson coverage or Shannon index (a). Principal Coordinates Analysis (PCoA) representing β-diversity on OTU level was analyzed (b). [file Image3.jpg]

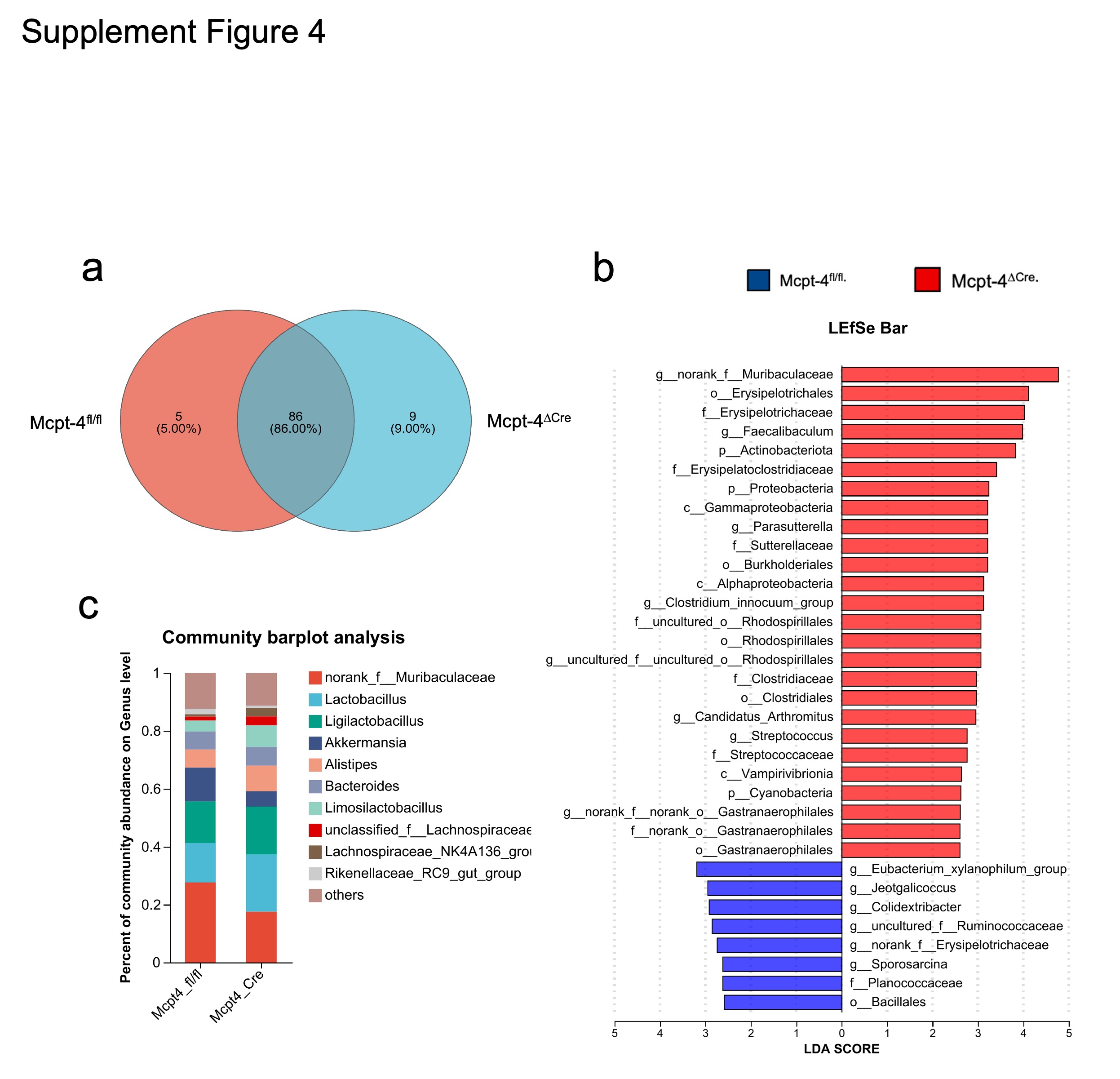

Supplement: Supplementary Figure 4 — The comparison of colonic microbiota of Mcpt-4fl/fl and Mcpt-4ΔCre mice. The microbiota data were further analyzed. The bacteria on genus level were counted between Mcpt-4fl/fl and Mcpt-4ΔCre groups (a). The linear discriminant analysis of effect size was calculated (b). The percent of community abundance of gut bacterial on genus levels was shown in (c). [file Image4.jpg]

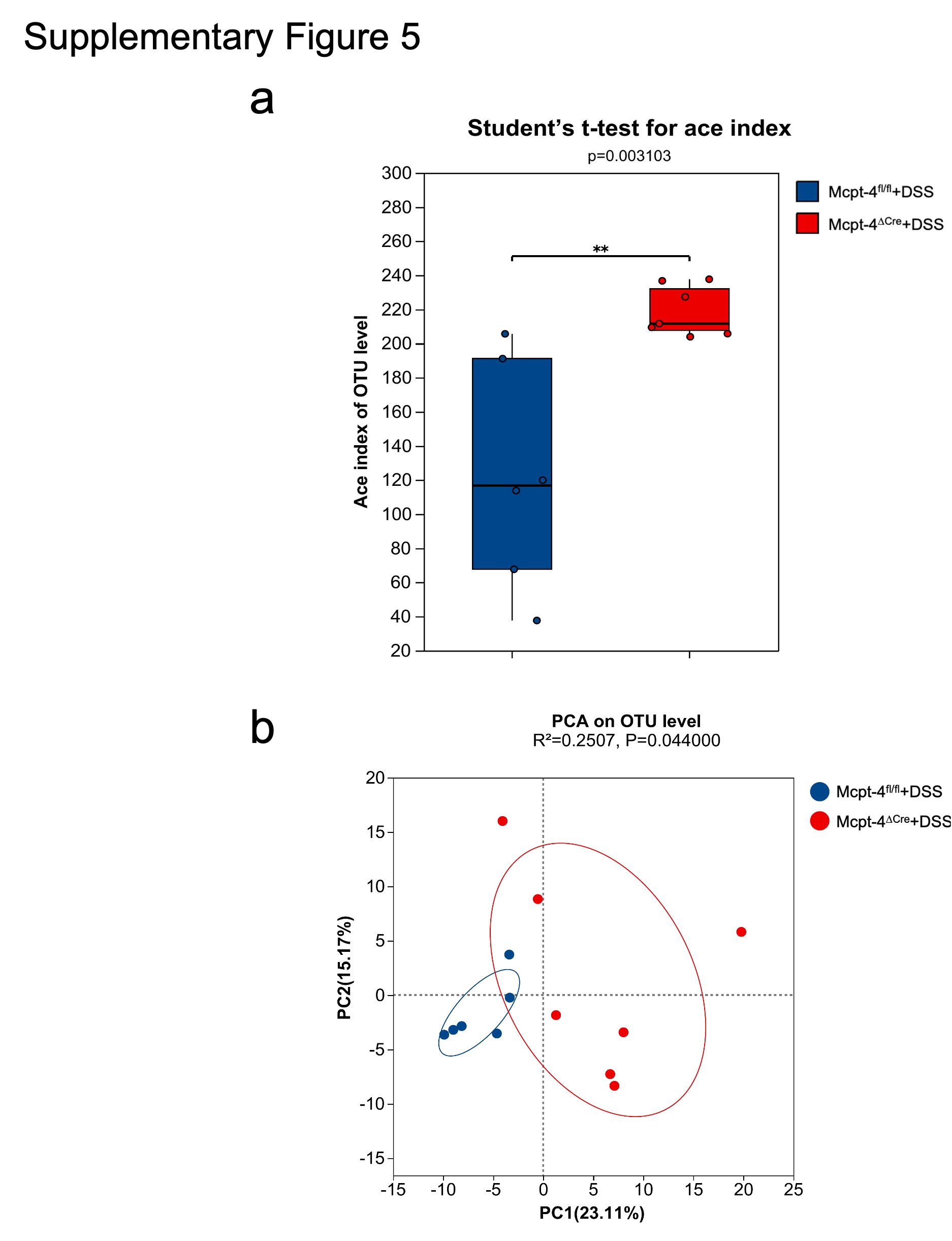

Supplement: Supplementary Figure 5 — The Mcpt-4 deficiency alters the composition of gut microbiota in colitis mice. Colon samples were collected from mice (n≧6 in each group) with colitis. 16s rRNA genes were sequenced with the microbial genomic DNA extracted from colon contents. A-diversity comparison between Mcpt-4fl/fl +DSS and Mcpt-4ΔCre +DSS groups using student’s t-test was represented by the Ace index (a). Principal Component Analysis (PCA) representing β-diversity on OTU level was analyzed (b). [file Image5.jpg]

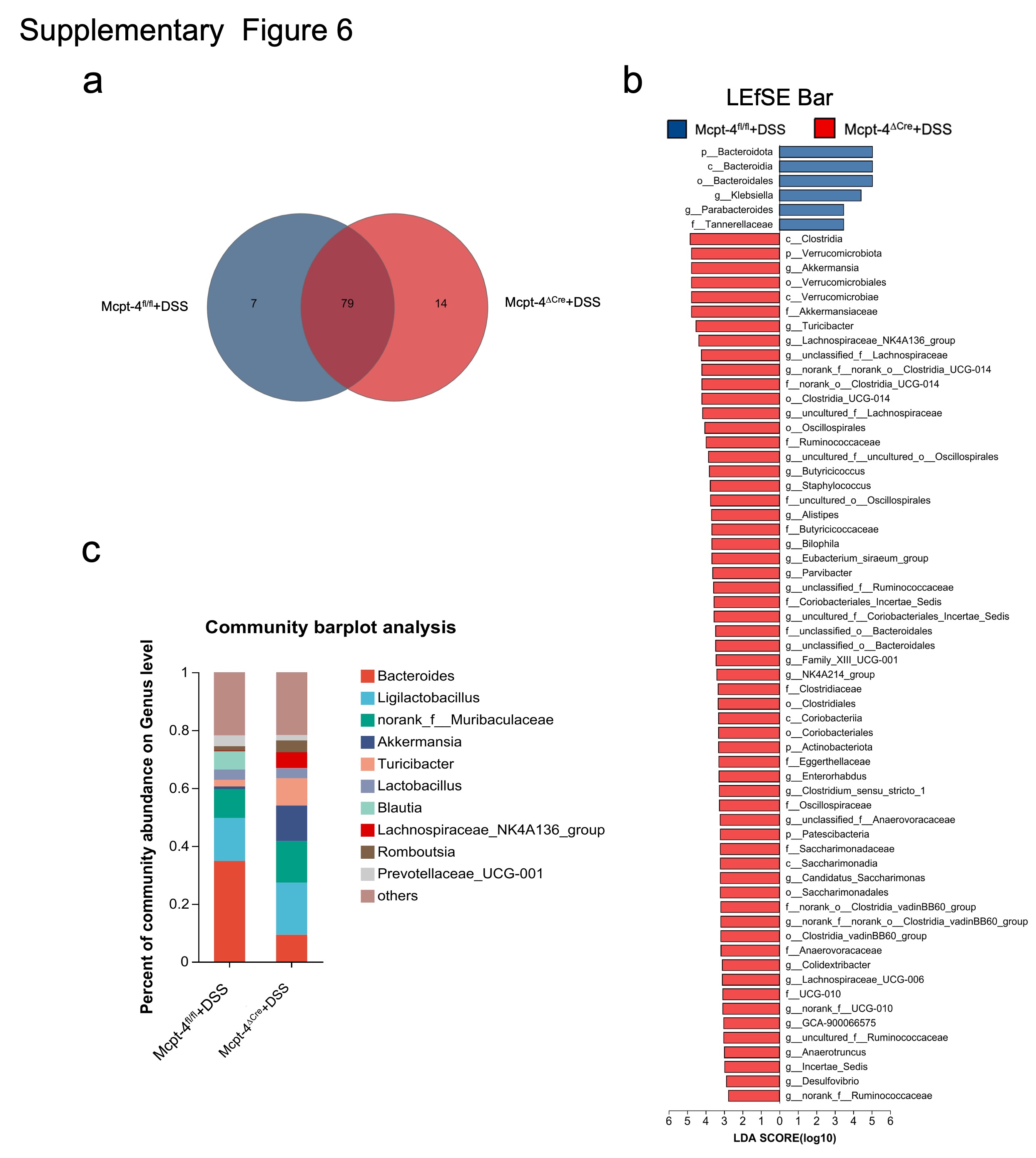

Supplement: Supplementary Figure 6 — The Mcpt-4 deficiency distorts the colonic microbiota of colitis mice. The microbiota data were further analyzed. The bacteria on OTU level were counted between Mcpt-4fl/fl +DSS and Mcpt-4ΔCre +DSS groups (a). The linear discriminant analysis of effect size was calculated (b). The percent of community abundance of gut bacterial on genus levels was shown in (c). [file Image6.jpg]

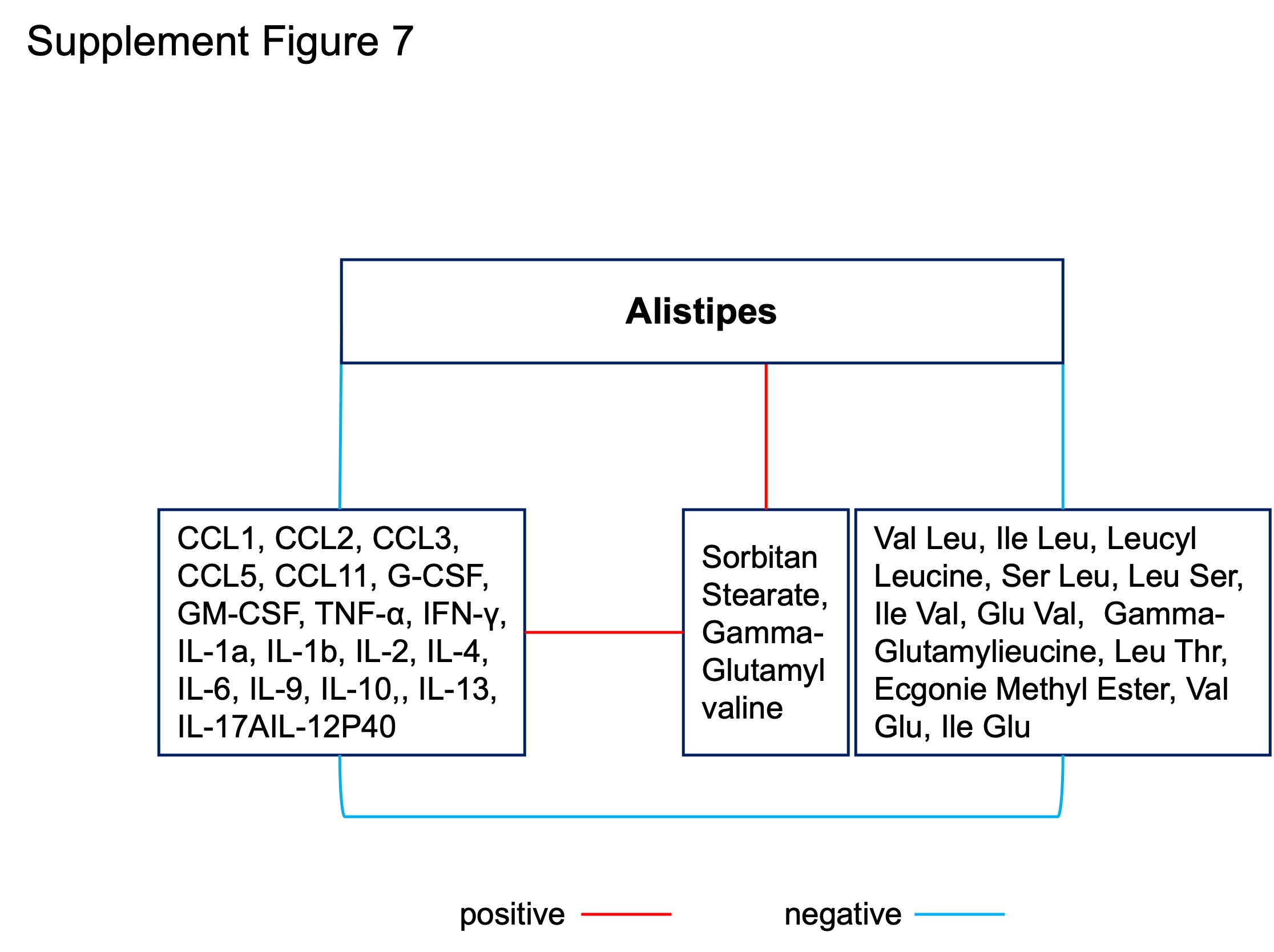

Supplement: Supplementary Figure 7 — Alistipes correlates with many cytokines and metabolites. Spearman correlation among colonic cytokines, microbiota (on genus level) and metabolite between Mcpt-4fl/fl +DSS and Mcpt-4ΔCre +DSS groups were analyzed. The red color indicates a positive correlation, while blue color indicates a negative correlation. [file Image7.jpg]
