# Supplementary material for: The deficiency of chymase mast cell protease 4 exacerbates dextran sulfate sodium salt-induced colitis in mice and is associated with altered microbiota and metabolome profiles
Source: Front Cell Infect Microbiol. 2025 Jul 8;15:1481927. doi: 10.3389/fcimb.2025.1481927 (PMC12279878; doi:10.3389/fcimb.2025.1481927)
Supplement: Supplementary file 8 [file Table1.docx]

UHPLC-Q Exactive HF-X

1. Metabolite Extraction

Solid sample：

50 mg solid sample was added to a 2 mL centrifuge tube and a 6 mm diameter grinding bead was added. 400 μL of extraction solution (methanol: water = 4:1 (v:v)) containing 0.02 mg/mL of internal standard (L-2-chlorophenylalanine) was used for metabolite extraction. Samples were ground by the Wonbio-96c ( Shanghai wanbo biotechnology co., LTD) frozen tissue grinder for 6 min (-10°C, 50 Hz), followed by low-temperature ultrasonic extraction for 30 min (5°C, 40 kHz). The samples were left at -20°C for 30 min, centrifuged for 15 min (4°C, 13000 g), and the supernatant was transferred to the injection vial for LC-MS/MS analysis.

2. Quality control sample

As a part of the system conditioning and quality control process, a pooled quality control sample (QC) was prepared by mixing equal volumes of all samples. The QC samples were disposed and tested in the same manner as the analytic samples. It helped to represent the whole sample set, which would be injected at regular intervals (every 5-15 samples) in order to monitor the stability of the analysis.

3. (UHPLC-MS/MS) analysis

The LC-MS/MS analysis of sample was conducted on a Thermo UHPLC-Q Exactive HF-X system equipped with anACQUITYHSS T3 column (100 mm × 2.1 mm i.d., 1.8 μm; Waters, USA) at Majorbio Bio-Pharm Technology Co. Ltd. (Shanghai, China). The mobile phases consisted of 0.1% formic acid in water:acetonitrile (95:5, v/v) (solventA) and 0.1% formic acid in acetonitrile:isopropanol:water (47.5:47.5, v/v) (solvent B). The flow rate was 0.40 mL/min and the column temperature was 40℃.

MS conditions:

The mass spectrometric data were collected using a Thermo UHPLC-Q Exactive HF-X Mass Spectrometer equipped with an electrospray ionization (ESI) source operating in positive mode and negative mode. The optimal conditions were set as followed: source temperature at

425℃ ; sheath gas flow rate at 50 arb;Aux gas flow rate at 13 arb; ion-spray voltage floating (ISVF) at -3500V in negative mode and 3500V in positive mode, respectively; Normalized collision energy , 20-40-60V rolling for MS/MS. Full MS resolution was 60000, and MS/MS resolution was 7500. Data acquisition was performed with the Data DependentAcquisition (DDA) mode.The detection was carried out over a mass range of 70-1050 m/z.

4. Data anlaysis

The pretreatment of LC/MS raw data was performed by Progenesis QI (Waters Corporation，Milford, USA) software, and a three-dimensional data matrix in CSV format was exported. The information in this three-dimensional matrix included: sample information, metabolite name and mass spectral response intensity. Internal standard peaks, as well as any known false positive peaks (including noise, column bleed, and derivatized reagent peaks), were removed from the data matrix, deredundant and peak pooled.At the same time, the metabolites were identified by searching database, and the main databases were the HMDB (http://www.hmdb.ca/), Metlin ( https://metlin.scripps.edu/) and Majorbio Database .

The data matrix obtained by searching database was uploaded to the Majorbio cloud platform (https://cloud.majorbio.com) for data analysis. Fistly, the data matrix was pre-processed, as follows: At least 80% of the metabolic features detected in any set of samples were retained. After filtering, for specific samples with metabolite levels below the lower limit of quantification, the minimum metabolite value was estimated and each metabolic signature was normalized to the sum.

To reduce the errors caused by sample preparation and instrument instability, the responseintensities of the sample mass spectrometry peaks were normalized using the sum normalization method, to obtain the normalized data matrix. Meanwhile, the variables of QC samples with relative standard deviation (RSD) > 30% were excluded and log10 logarithmicized, to obtain the final data matrix for subsequent analysis.

Then, the R package “ropls”(Version 1.6.2) was used to perform principal component analysis (PCA) and orthogonal least partial squares discriminant analysis (OPLS-DA), and 7-cycle interactive validation evaluating the stability of the model. The metabolites with VIP>1, p<0.05 were determined as significantly different metabolites based on the Variable importance in the projeciton (VIP) obtained by the OPLS-DAmodel and the p-value generated by student’s t test.

Differential metabolites among two groups were mapped into their biochemical pathways through metabolic enrichment and pathway analysis based on KEGG database (http://www.

genome.jp/kegg/). These metabolites could be classified according to the pathways they involved or the functions they performed. Enrichment analysis was used to analyze a group of metabolites in a function node whether appears or not. The principle was that the annotation analysis of a single metabolite develops into an annotation analysis of a group of metabolites. Python packages “scipy.stats” (https://docs.scipy.org/doc/scipy/ ) was used to perform enrichment analysis to obtain the most relevant biological pathways for experimental treatments.
